# Bioactive Profiling and Evaluation of Anti-Proliferative Potential of *Salvadora persica* Bark Extract in Triple-Negative Breast Cancer Cells: An In Vitro and Computational Analysis

**DOI:** 10.3390/life16060943

**Published:** 2026-06-03

**Authors:** Abrar Turki, Md. Abul Barkat, Yasmin Basheer Ahmed, Harshita Barkat, Raghad Rashed Alotaibi, Shivbrat Upadhyay, Juveriya Israr, Sahabjada Siddiqui

**Affiliations:** 1 Clinical Nutrition Department, College of Applied Medical Sciences, University of Hafr Al Batin, Hafr Al Batin 39524, Eastern Province, Saudi Arabia; aamturki@uhb.edu.sa (A.T.); ybahmed@uhb.edu.sa (Y.B.A.); 2 Department of Pharmaceutics, College of Pharmacy, University of Hafr Al Batin, Hafr Al Batin 39524, Eastern Province, Saudi Arabia; habarkat@uhb.edu.sa (H.B.); s2221003475@uhb.edu.sa (R.R.A.); 3 Department of Biotechnology, Era’s Lucknow Medical College and Hospital, Era University, Lucknow 226003, India; pranjal2050@gmail.com (S.U.); juveriyaisrar2016@gmail.com (J.I.); sahabjada@erauniversity.in (S.S.)

**Keywords:** *Salvadora persica* bark, UHPLC analysis, anticancer, TNBC, in silico analysis

## Abstract

**Background:** *Salvadora persica* (*S. persica*), commonly known as ‘Miswak,’ has been used in ethnotraditional applications since ancient times. This study was formulated to examine bioactive phenolics and flavonoids from the hydroethanolic extract of *S. persica* bark, anticancer activity, and in silico binding interaction analysis with key therapeutic targets of triple-negative breast cancer (TNBC) cells. **Methods:** UHPLC was used to identify the phytochemicals in *S. persica* bark extract. Cell death was analyzed by MTT assay in TNBC MDA-MB-231 and MDA-MB-468 cells. Moreover, cellular apoptosis, ROS generation, MMP, and cell cycle checkpoints were also carried out. AutoDock Tools 1.5.7 and PyRx 0.8 tools were used for molecular binding interaction analysis. **Results:** Phytochemical analysis revealed the presence of total phenolic and total flavonoid content of 26.90 ± 0.46 μg GAE/mg and 54.51 ± 0.42 μg QE/mg of bark extract, respectively. UHPLC analysis confirmed the presence of fumaric acid, chlorogenic acid, rutin, and quercetin in the extract. *S. persica* significantly reduced cell viability of MDA-MB-231 and MDA-MB-468 cells with an IC_50_ value of 144 and 128 μg/mL, respectively. *S. persica* extract elevated ROS generation, loss of MMP, late apoptosis induction, and G2/M-phase cell cycle arrest, while it did not show any significant effect against normal kidney Vero cells. Molecular docking studies revealed that rutin showed strong binding affinity towards EGFR with B.E. = −9.8 and −9.5 Kcal/mol; FGFR1 with B.E. = −7.4 and −7.5 Kcal/mol; FGFR4 with B.E. = −7.5 and −7.9 Kcal/mol; and csGRP78 with B.E. = −9.0 and −9.3 Kcal/mol, using Autodock Vina and PyRx tools, respectively. SwissADME and drug-likeness analysis confirmed acceptable drug-like characteristics and favorable pharmacokinetic profiles of the identified molecules. **Conclusions:** This study highlighted the potential of phytochemicals from *S. persica* bark as promising compounds for the development of novel anticancer therapeutics.

## 1. Introduction

Breast cancer is the leading cancer affecting women across the globe and continues to be a major cause of cancer-related deaths. According to GLOBOCAN 2022 estimates, about 2.3 million new cases and more than 665,000 deaths were reported globally, underscoring its significant impact on public health [1]. In India, breast cancer has overtaken cervical cancer to become the most prevalent malignancy among women, contributing to nearly one-quarter of all female cancer cases. Its incidence is steadily increasing, especially in urban populations and among younger women. Although improvements in early detection and targeted treatments have been achieved, the mortality rate remains high, largely due to delayed diagnosis, tumor diversity, and the development of resistance to therapy [2]. Breast cancer is a biologically diverse disease and is commonly categorized into hormone receptor-positive (ER/PR+), HER2-enriched, and triple-negative breast cancer (TNBC) subtypes. TNBC is characterized by the absence of estrogen receptor (ER), progesterone receptor (PR), and human epidermal growth factor receptor-2 (HER2) expression, and it accounts for roughly 15–20% of all breast cancer cases [3]. TNBC is an aggressive subtype characterized by a high likelihood of recurrence and metastasis, and it is more frequently observed in younger women. In contrast to hormone receptor and HER2-positive breast cancers, TNBC lacks clearly defined molecular targets, leaving surgery and systemic chemotherapy as the primary treatment approaches. Although therapies such as immune checkpoint inhibitors and PARP inhibitors have demonstrated benefits in certain patient groups, overall survival remains limited due to rapid disease progression, resistance to chemotherapy, and treatment-related toxicity [4].

Recent evidence indicates that dysregulation of growth factor receptors, particularly epidermal growth factor receptor (EGFR) and fibroblast growth factor receptors (FGFRs), as well as stress-related chaperone proteins such as cell surface GRP78, contributes significantly to the progression of TNBC [5,6]. These molecules are involved in promoting tumor growth, enhancing cell survival, and facilitating metastasis, making them attractive targets for therapeutic intervention; therefore, these membrane receptors were selected in the current study. Many of the currently available targeted therapies are expensive and often associated with adverse effects, underscoring the need for safer and more cost-effective treatment options. Natural products have long been recognized as a valuable source of anticancer agents, with a substantial proportion of current drugs originating from natural compounds. Phytochemicals, including flavonoids, phenolic acids, and alkaloids, have shown promising anticancer activity through multiple mechanisms. These include induction of apoptosis via disruption of MMP, ROS generation, arrest of the cell cycle, and regulation of critical signaling pathways such as PI3K/Akt, MAPK, and EGFR [7]. Notably, many of these compounds exhibit selective toxicity toward cancer cells while minimizing damage to normal tissues, highlighting their potential as effective and safer therapeutic candidates.

*Salvadora persica* (*S. persica*), commonly referred to as miswak or the “bitter stick,” has a long history of use in traditional medicine systems across the Middle East, Africa, and the Indian subcontinent. Its roots and twigs have been extensively utilized as natural chewing sticks for maintaining oral hygiene, supported by evidence of antimicrobial, anti-inflammatory, and plaque-inhibiting properties [8,9]. Within Ayurvedic and Unani medicinal practices, *S. persica* is traditionally recommended for the treatment of dental caries, gingivitis, halitosis, and other periodontal conditions [10]. In addition to its role in oral health, preparations made from its bark and roots have been used as mild laxatives, digestive aids, and remedies for various gastrointestinal complaints. Traditional applications further include the management of skin infections, enhancement of wound healing, and relief from rheumatic and respiratory disorders. Phytochemical studies have revealed that these therapeutic effects are associated with the presence of diverse bioactive compounds, including flavonoids, alkaloids, sterols, and phenolic acids. These constituents are known to exhibit significant antimicrobial, antioxidant, and cytotoxic activities [11].

The extensive traditional use of *S. persica*, together with growing experimental evidence, highlights its promise as a valuable source of bioactive agents for future biomedical and pharmacological research. *S. persica* root extracts have shown anticancer activity against hepatocellular carcinoma (HepG2) cells and tongue squamous cell (HNO 97) carcinoma [12,13], while fruit extracts have been evaluated against MCF-7 breast cancer cells [14]. However, research on the underlying mechanisms of *S. persica* bark extract is limited, especially in the context of triple-negative breast cancer cells. Moreover, comprehensive studies that integrate phytochemical characterization with in silico molecular docking against key therapeutic targets in TNBC are still limited. To our knowledge, no study has integrated UHPLC-based phytochemical profiling with in vitro mechanistic apoptosis analysis and multi-target in silico docking of *S. persica* bark extract phytoconstituents against TNBC cells. In this study, the hydroethanolic extract of *S. persica* was used for phytochemical characterization, in vitro anticancer analysis against TNBC cells, and in silico binding interaction analysis with multiple therapeutic receptors of TNBC. *S. persica* bark extract potentially exerts a combined effect, as its identified phenolic and flavonoid components can interact with therapeutic targets and induce apoptosis through ROS-mediated mechanisms.

## 2. Materials and Methods

### 2.1. Reagents and Chemicals

DMEM/F-12 medium, fetal bovine serum (FBS), penicillin–streptomycin antibiotics, DCFH-DA, and Rhodamine-123 dyes were obtained from Sigma-Aldrich (St. Louis, MO, USA). DAPI dye and MTT reagent were sourced from HiMedia (Mumbai, India). The Annexin V-FITC apoptosis detection kit was purchased from BioVision (Milpitas, CA, USA). All chemicals and reagents used in the study were of analytical grade.

### 2.2. Collection of Plant Materials and Identification

Fresh plant *S. persica* was collected from Maharaj Ganj, Raebareli, Uttar Pradesh, India, in August 2025. Plant material (Accession No. IU/PHAR/HRB/25/08) was identified, and the specimen was deposited at the Department of Pharmacognosy and Phytochemistry, Integral University, Lucknow, India.

### 2.3. S. persica Bark Extract Preparation

The bark material was separated from roots and washed with tap water, followed by double-distilled water, and then shade-dried for 2 weeks. The bark was mechanically chopped into coarse particles and then coarsely ground in a mixer grinder (Bajaj Rex, Mumbai, India). Hydro-ethanol (75%) was used to extract the phytochemicals through the percolation method, and incubated for about 2 days at room temperature. The solvent extract was filtered through Whatman No. 1 filter paper (125 mm). The obtained *S. persica* bark extract was evaporated to dryness under reduced pressure at 45 °C using a Rotavapor evaporator (Buchi Rotavapor R-205, Allschwil, Switzerland). The resulting extract was further concentrated in a water bath to produce a semi-solid paste, which was then kept in an airtight container at 4 °C for use in experiments.

### 2.4. Estimation of Total Phenolic Contents (TPC)

The TPC was determined by the Folin–Ciocalteu (FC) reagent with slight modification as per standard protocol [15]. The methanolic extract of *S. persica* (1 mg/mL) was reacted with 1.5 mL (10%) FC reagent and 3.0 mL of (7.5%) sodium carbonate (Na_2_CO_3_). The reacted solution was incubated at 37 °C for one hour. Absorbance of the solution was measured on a microplate reader (Thermo Fisher Scientific, Mumbai, India) at 760 nm. The gallic acid standard curve was used for the calculation of total phenol content, and the unit is μg GAE/mg extract (Figure S1).

### 2.5. Estimation of Total Flavonoid Contents (TFC)

The TFC was determined by the aluminum chloride (AlCl3) colorimetric method with slight modification as previously reported by Chandra et al. [15]. The hydro-ethanolic extract of *S. persica* (1 mg/mL) was mixed with 200 μL-10% AlCl3, 200 μL-1 M potassium acetate (CH_3_COOK), 3.8 mL of ultrapure water, and incubated at 37 °C for one h. The absorbance was taken on a Thermo Fisher microplate reader at 510 nm. Estimation of total flavonoid contents was calculated with the help of a quercetin standard curve, and the measuring unit was μgQE/mg extract (Figure S2).

### 2.6. Ultra-High Performance Liquid Chromatography (UHPLC) Analysis

Phytochemical analysis was performed using UHPLC Nexera series (Shimadzu, Kyoto, Japan) equipped with the quaternary solvent delivery pump, autosampler, fluorescence detector, and photodiode array detector (Shimadzu). All the solvents were HPLC-grade, purchased from E. Merck (Mumbai, India). The optimum chromatographic separation for flavonoids and phenolic acids was optimized on a reverse-phase column (Thermoscientific, Waltham, MA, USA, 2.6 μm, 2.1 × 150 mm). The chromatographic separation of flavonoids was optimized using water with 0.1% formic acid and methanol with a gradient program. The column temperature was set at 40 °C, and the injection volume was 5.0 μL. For phenols, water with 0.1% formic acid and acetonitrile with 0.1% formic acid were taken as the mobile phase. The column temperature was set at 40 °C, and the injection volume was 4.0 μL.

### 2.7. Cell Line and Culture

Normal kidney epithelial Vero cell lines and TNBC cells MDA-MB-231 and MDA-MB-468 were bought from the NCCS, Pune, India. In 25 cc tissue culture flasks, cells were grown in DMEM: F12 (1:1) media supplemented with 10% heat-inactivated FBS, 2 mM L-glutamine, 1% antibiotic solution in an incubator (Model-371, Thermo Scientific, Waltham, MA, USA) at 37 °C and 5% CO_2_.

### 2.8. MTT Assay

*S. persica* extract’s potential to stop cell growth against Human TNBC MDA-MB-231, MDA-MB-468, and normal kidney epithelial Vero cell line was measured using the MTT test as a standard procedure [16]. All cell lines were seeded at a density of 1 × 10^4^ cells/mL in 96-well microtiter culture plates overnight. Initially, *S. persica* bark extract stock was prepared in a culture medium and was further diluted in the same media to the concentrations of 50, 100, 200, 300, and 400 µg/mL to treat cultured cells for 24 h. MTT dye was added to each well, and the developed formazan crystal was dissolved in DMSO, followed by absorbance reading at 550 nm using an ELISA plate reader (Bio-Rad PW41, Hercules, CA, USA). The IC50 values were determined using regression curve analysis through MS Excel. Inverted phase contrast microscopy (Nikon Eclipse TS100, Tokyo, Japan) was used to detect structural alterations at the cellular level.

### 2.9. Nuclear Condensation Assay

Three effective concentrations of 100 µg/mL (low dose; LD, dose below IC_50_), 144 µg/mL (IC_50_ value), and 200 µg/mL (high dose; HD, dose above IC_50_) of *S. persica* bark extract were used to investigate the apoptosis-inducing potential of *S. persica* bark extract in a dose-dependent manner. As previously mentioned, nuclear condensation was evaluated by DAPI staining [16]. In short, cells were fixed for ten minutes with 4% paraformaldehyde after being washed with PBS. After fixation, cells were permeabilized using a permeabilization buffer and stained with DAPI dye. An inverted fluorescent phase contrast microscope (Zeiss AxioVert 135, New York, NY, USA) was used to capture images of stained cells.

### 2.10. Annexin V-FITC Double Staining for Apoptosis Investigation

The proportions of viable, dead, and apoptotic MDA-MB-231 cells at LD, IC50, and HD were analyzed using flow cytometry (FACS Lyric, BD Biosciences, Franklin Lakes, NJ, USA) with an Annexin V-FITC apoptosis detection kit. Briefly, cells were seeded at a density of 1 × 10^6^ cells/well of a 6-well plate and treated with *S. persica* bark extract at the specified concentrations for 24 h. Cells were pelleted after treatment and then resuspended in binding buffer. After staining the cell suspension with 5 µL of Annexin V-FITC and 5 µL of propidium iodide (PI), it was incubated for 15 min at 25 °C in the dark. Subsequently, samples were analyzed by flow cytometry to quantify apoptotic cell populations.

### 2.11. Analysis of the Intracellular ROS Level

ROS generation was measured using DCFH-DA staining followed by flow cytometric analysis, as previously described [16]. After harvesting the treated and control cells, they were washed with PBS and incubated for 20 min at 37 °C with 10 μM DCFH-DA in PBS. Cells were then washed twice with PBS to remove excess dye and subjected to flow cytometry for ROS measurement.

### 2.12. Evaluation of Mitochondrial Membrane Potential (MMP, ΔΨm)

As described previously [16], changes in MMP were assessed using the fluorescent probe Rhodamine-123 (Rh-123) by flow cytometry. Briefly, cells were incubated with 10 μM Rh-123 for 30 min in the dark, followed by two washes with PBS. The cells were then resuspended in 500 μL of PBS and subjected to flow cytometric analysis.

### 2.13. Analysis of Cellular DNA Content

Cells were seeded at a density of 1 × 10^6^ cells/well of a 6-well plate and treated with *S. persica* bark extract at LD, IC_50_, and HD for 24 h. Following a cold PBS wash, the cultured cells were fixed for two h at −20 °C in 70% ethanol. RNase A (10 mg/mL) was applied to lyse RNA from cells, and they were then stained with PI for 30 min at RT in the dark. Subsequently, cell cycle distribution and DNA content were analyzed using flow cytometry, following the procedure described earlier [16]. Data were analyzed with the help of Cell Quest Pro V 3.2.1 software (Becton Dickinson, Franklin Lakes, NJ, USA).

### 2.14. In Silico Analysis

#### 2.14.1. Ligand Retrieval and Preparation

Ligands corresponding to the identified bioactive compounds (Rutin-PubChem CIDs: 5280805; quercetin-5280343; chlorogenic acid-1794427; fumaric acid-444972) were retrieved in 3D SDF format from the PubChem database. Structures underwent energy minimization in Avogadro 1.2.0 employing the MMFF94 force field (1000 steps steepest descent, convergence criterion: 0.01 kcal/mol·Å RMS gradient) to optimize geometries. For AutoDock Vina docking, ligands were processed in AutoDockTools 1.5.7 (MGLTools): polar hydrogens were added, Gasteiger-Marsili partial charges were computed, non-polar hydrogens merged to polar atoms, and rotatable bonds were identified via the torsion tree algorithm. Files were exported as PDBQT with explicit torsional degrees of freedom. For PyRx docking, ligand preparation followed an identical workflow within AutoDockTools 1.5.7 to ensure compatibility, generating PDBQT files directly importable into PyRx 0.8. Structural integrity and charge neutrality were verified in both instances using Open Babel feature for interconversion and validation.

#### 2.14.2. Protein Retrieval and Preparation

Crystal structures of target proteins were obtained from the Protein Data Bank (RCSB PDB): EGFR/ErbB1 (PDB ID: 1IVO), FGFR1 (PDB ID: 1EVT), FGFR4 (PDB ID: 4TYE), and csGRP78 (PDB ID: 3IUC). Structures were refined in BIOVIA Discovery Studio 2022: removal of crystallographic waters, co-crystallized ligands, and heteroatoms.

#### 2.14.3. Molecular Docking: AutoDock Vina

While using AutoDock Vina for molecular docking, receptor and ligand files in PDBQT format were specified in a configuration file with the following parameters: exhaustiveness, num_modes, and energy range kcal/mol. Grid boxes were centered on the active sites of target proteins with dimensions in Å spacing (coordinates detailed in Table 1). Output poses were ranked by predicted binding free energy (kcal/mol); the lowest-energy conformation for each ligand–protein pair was selected for interaction analysis. Intermolecular interactions, including hydrogen bonds, hydrophobic contacts, π-π stacking, and π–cation interactions, were visualized and quantified, and the Protein–Ligand Interaction through BIOVIA Discovery Studio 2022.

#### 2.14.4. Molecular Docking: PyRx

Virtual high-throughput screening was conducted using PyRx 0.8, which integrates AutoDock Vina as its docking engine. Prepared PDBQT files for proteins and ligands were imported into PyRx; docking grids were defined identically to AutoDock Vina parameters (exhaustiveness = 8, grid dimensions in Å spacing. The Vina wizard facilitated batch docking of all four ligands against each target protein. Binding poses were ranked by binding affinity scores; consensus top-ranking poses were prioritized. Two-dimensional ligand interaction diagrams were generated natively in PyRx OpenGL, highlighting key residue interactions for validation against crystallographic inhibitors. Sites were defined by grid boxes centered on catalytic/binding residues, with coordinates as shown in Table S1.

#### 2.14.5. SwissADME Analysis

Physicochemical, pharmacokinetic, and drug-likeness properties were predicted using the SwissADME webserver (http://www.swissadme.ch). Input structures (SMILES from PubChem) yielded: lipophilicity (LogP WLOGP/iLOGP), water solubility (LogS), molecular descriptors (MW, rotatable bonds, TPSA), drug-likeness rules (Lipinski, Muegge, Veber, Ghose, Egan), blood–brain barrier permeation, gastrointestinal absorption (BOLED-Egg plot), P-glycoprotein substrate status, and CYP450 inhibition profiles (2C9, 2D6, 3A4).

#### 2.14.6. Toxicity Prediction

Toxicity risks (mutagenicity, tumorigenic potential, irritancy, reproductive toxicity) and drug score were forecasted using Osiris Property Explorer/DataWarrior. QSAR models computed parameters including cLogP, LogS, molecular weight, and toxicity flags. Drug-likeness score integrated physicochemical properties and toxicity predictions, with scores >0.5 denoting favorable candidates.

#### 2.14.7. Statistical Analysis

The mean ± SD of three separate experiments was used to express the experimental findings. One-way ANOVA and Dunnett’s multiple comparison test using GraphPad Prism (v5.01) were used to establish statistical significance; *p* < 0.05 was deemed statistically significant.

## 3. Results

### 3.1. Estimation of Total Phenolic Contents (TPC)

The TPC of *S. persica* bark extract was determined by using gallic acid as a standard. The result was demonstrated as gallic acid equivalents per gram of dry weight of extract (μg GAE/mg) with the help of a gallic acid standard curve and a correlation coefficient R^2^ = 0.9806 (Figure S1). Significant amounts of phenol content, 26.90 ± 0.46 μg GAE/mg, were present in bark extract.

### 3.2. Estimation of Total Flavonoid Contents (TFC)

The estimation of the TFC of *S. persica* bark extract was determined using quercetin as a standard. The result was demonstrated as quercetin equivalent per gram of dry weight of extract (μg QE/mg) with the help of the quercetin standard curve and correlation coefficient R^2^ = 0.988 (Figure S2). Significant amounts of flavonoid content, 54.51 ± 0.42 μg QE/mg, were present in the bark extract.

### 3.3. Phytochemical Characterization of the Extract by UHPLC

**Figure 1:**
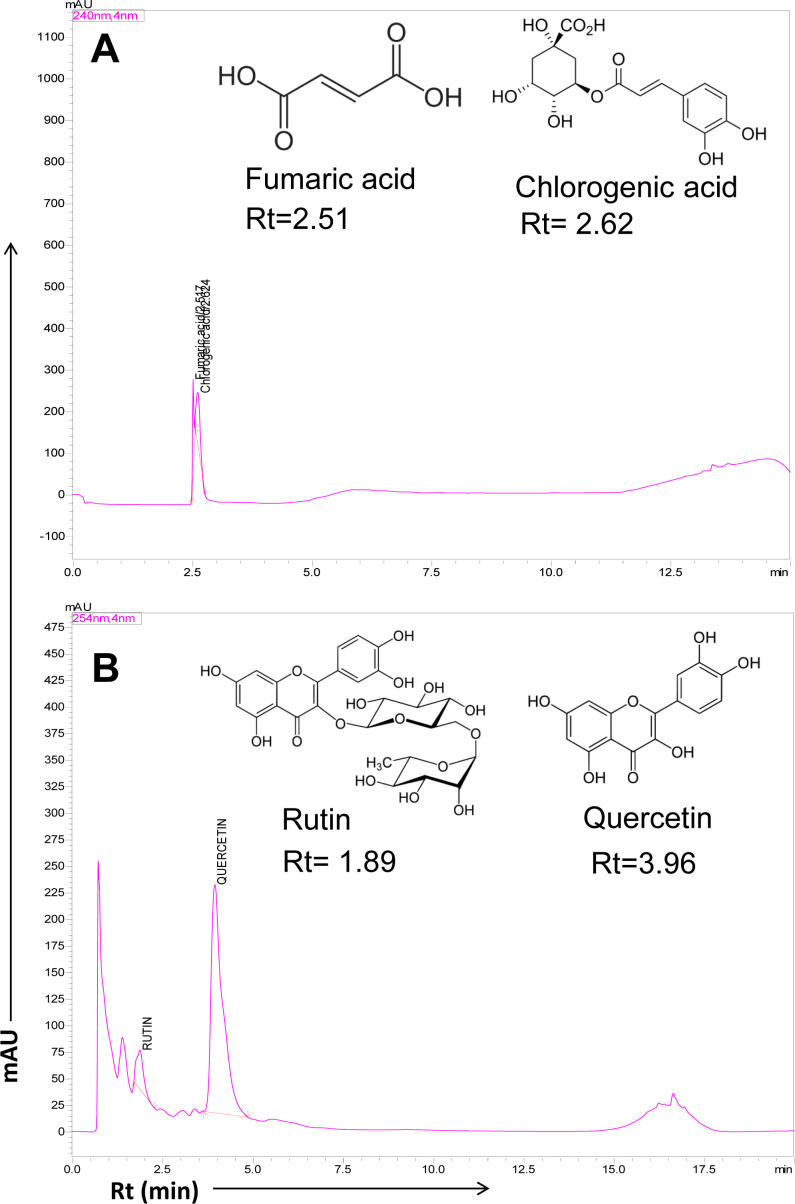
Chromatogram of phenolic and flavonoid compounds in the *S. persica* bark extract using UHPLC analysis (**A**) UHPLC chromatogram showing the presence of phenolic compound fumaric acid (Rt = 2.51 min) and chlorogenic acid (Rt = 2.62 min). (**B**) UHPLC chromatogram showing the presence of flavonoid compounds rutin (Rt = 1.89 min) and quercetin (Rt = 3.96 min). The X-axis shows the retention time (Rt) in minutes, while the Y-axis represents the signal intensity in milli-absorbance units (mAU).

UHPLC was employed to identify the bioactive constituents present in the *S. persica* bark extract. Analysis of the hydroethanolic bark extract revealed the presence of both phenolic and flavonoid compounds, as illustrated in the chromatograms (Figure 1A,B). The UHPLC profile for phenolics displayed two distinct peaks, corresponding to fumaric acid (retention time, Rt = 2.51 min) and chlorogenic acid (Rt = 2.62 min). In addition, two flavonoids were identified, namely rutin and quercetin, with retention times of 1.89 min and 3.96 min, respectively.

### 3.4. Morphological Analysis and Cell Viability Assay of S. persica Bark Extract Against TNBC Cells

**Figure 2:**
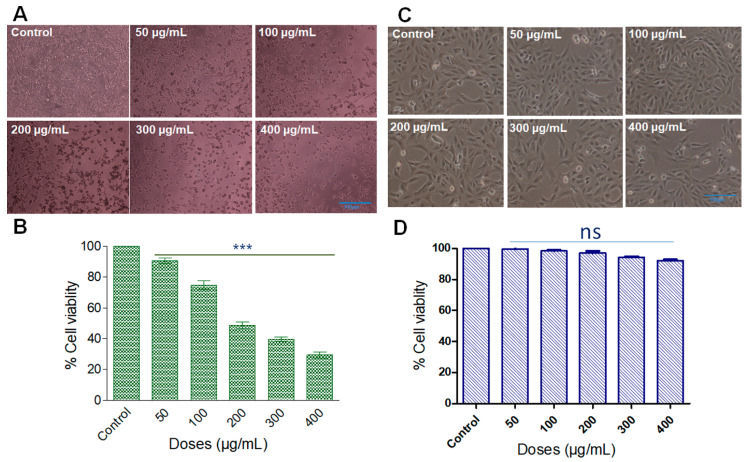
Cytotoxic test of *S. persica* bark extract on human TNBC breast cancer MDA-MB-231 and normal Vero cells. (**A**) Analysis of morphological changes in MDA-MB-231 cells subjected to bark extract, the concentrations ranging from 50 to 400 µg/mL, under an inverted microscope; (**B**) cytotoxicity of bark extract was quantified as the percentage cell viability of MDA-MB-231 cells at 24 h; (**C**) analysis of morphological changes in Vero cells subjected to bark extract under inverted microscope; (**D**) cytotoxicity of bark extract was quantified as the percentage cell viability of Vero cells at 24 h. Values from a minimum of three independent experiments are shown as Mean ± SD, with *** *p* < 0.001 in comparison to the control.

TNBC cells were treated with increasing concentrations of *S. persica* bark extract (50–400 µg/mL) for 24 h. The IC_50_ values for MDA-MB-231 and MDA-MB-468 cells were calculated to be 144 and 128 µg/mL, respectively (Figure 2 and Figure S3). Considering the highly invasive and aggressive tumor characteristics of MDA-MB-231, which make it a widely accepted model in anticancer research, a detailed study was conducted using this cell line. Compared with untreated MDA-MB-231 cells, which displayed a typical adherent, uniform morphology, treated cells showed marked structural alterations, becoming rounded and detached (Figure 2A). Cell viability results (Figure 2B) demonstrated a clear dose-dependent decline following *S. persica* bark extract exposure. The findings indicate that the extract effectively inhibits the proliferation of TNBC cells. In contrast, Vero normal cells did not exhibit significant morphological changes or a substantial loss of viability after treatment (Figure 2C,D). Only a small fraction of cells showed detachment at higher concentrations following 24 h of exposure. Based on the IC_50_ value obtained for MDA-MB-231 cells, three concentrations were selected for subsequent in vitro cell death studies: low dose (LD, 100 µg/mL), IC_50_ dose (144 µg/mL), and high dose (HD, 200 µg/mL).

### 3.5. Nuclear Condensation Assay

**Figure 3:**
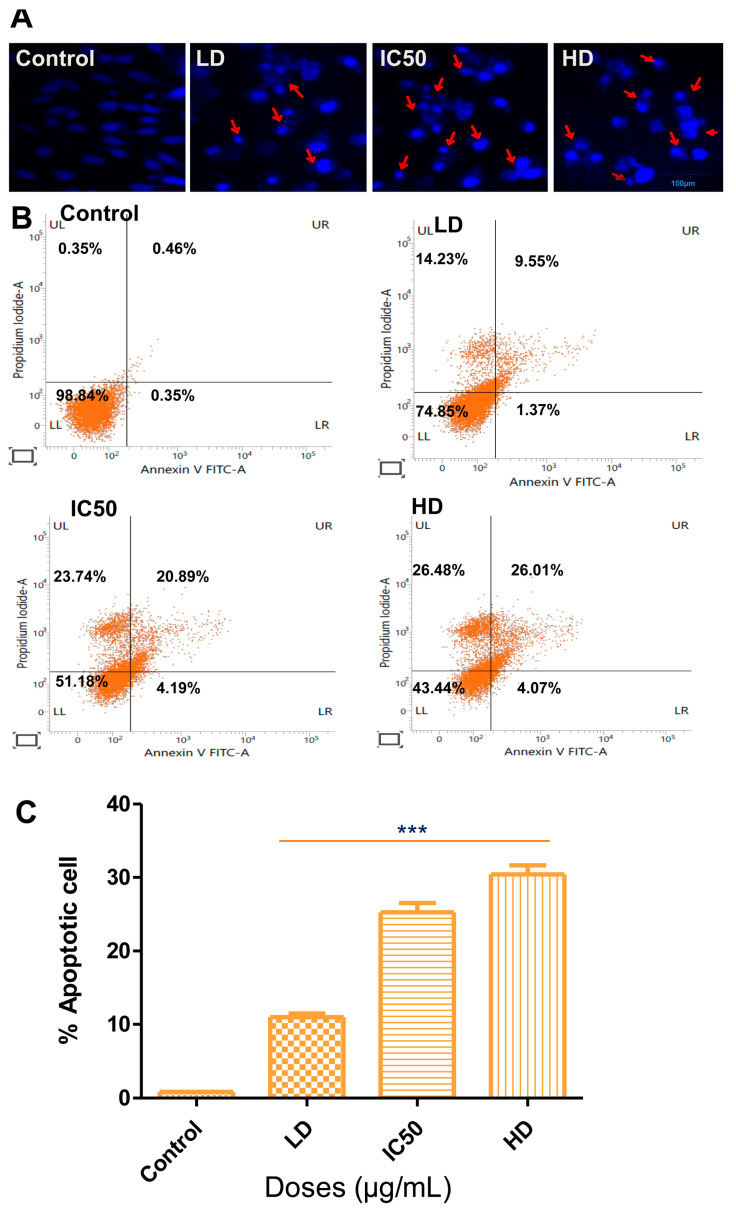
Apoptosis-inducing activity of *S. persica* bark extract. (**A**) Photomicrographs show the nuclear condensation in TNBC-treated cells stained with DAPI dye at low dose (LD = 100 μg/mL), IC_50_ (=144 μg/mL), and high dose (HD = 200 μg/mL) of bark extract after 24 h. Nuclear condensation is indicated by arrows. Scale bar = 100 μm. (**B**) Bark extract-mediated induction of apoptosis early and late in MDA-MB-231 cells. Flow cytometry analysis after 24 h of treatment using annexin V/FITC & PI double stain. Representative figures showing the population of viable (annexin V− PI−), early apoptotic (annexin V+ PI−), late apoptotic (annexin V+ PI+), and necrotic (annexin V− PI+) cells. (**C**) Graph showing the total percentage of total apoptotic cells analyzed by the flow cytometer. *** *p* < 0.001 when compared to the control group (*n* = 3).

Microscopic examination of chromatin structure in MDA-MB-231 cells exposed to *S. persica* bark for 24 h revealed pronounced nuclear condensation compared with untreated control cells. The extent of chromatin condensation increased in a dose-dependent manner following *S. persica* bark treatment, with the most prominent effect observed at high dose (Figure 3A).

### 3.6. Apoptosis Quantification

To assess the quantitative estimation of apoptosis induction, TNBC cells were further analyzed using an Annexin V-FITC apoptosis detection kit (BioVision, Milpitas, CA, USA). Untreated control cells showed a viability of 98.84%, indicating a predominantly healthy cell population. Treatment with the *S. persica* bark extract resulted in a dose-dependent increase in apoptotic cell death. At the low dose (LD), early apoptotic populations were 1.37% while late apoptotic/secondary necrotic cells were 23.78%. At the IC_50_ concentration, early apoptosis increased to 4.19%, whereas late apoptotic/secondary necrotic cells increased to 44.63%. At the high dose (HD), early apoptotic cells accounted for 4.07%, while late apoptotic/secondary necrosis was 52.49% (Figure 3B). Collectively, bark extract demonstrated a pronounced ability to induce apoptosis in human TNBC MDA-MB-231 cells (Figure 3C).

### 3.7. S. persica Bark Induces Intracellular ROS Generation

**Figure 4:**
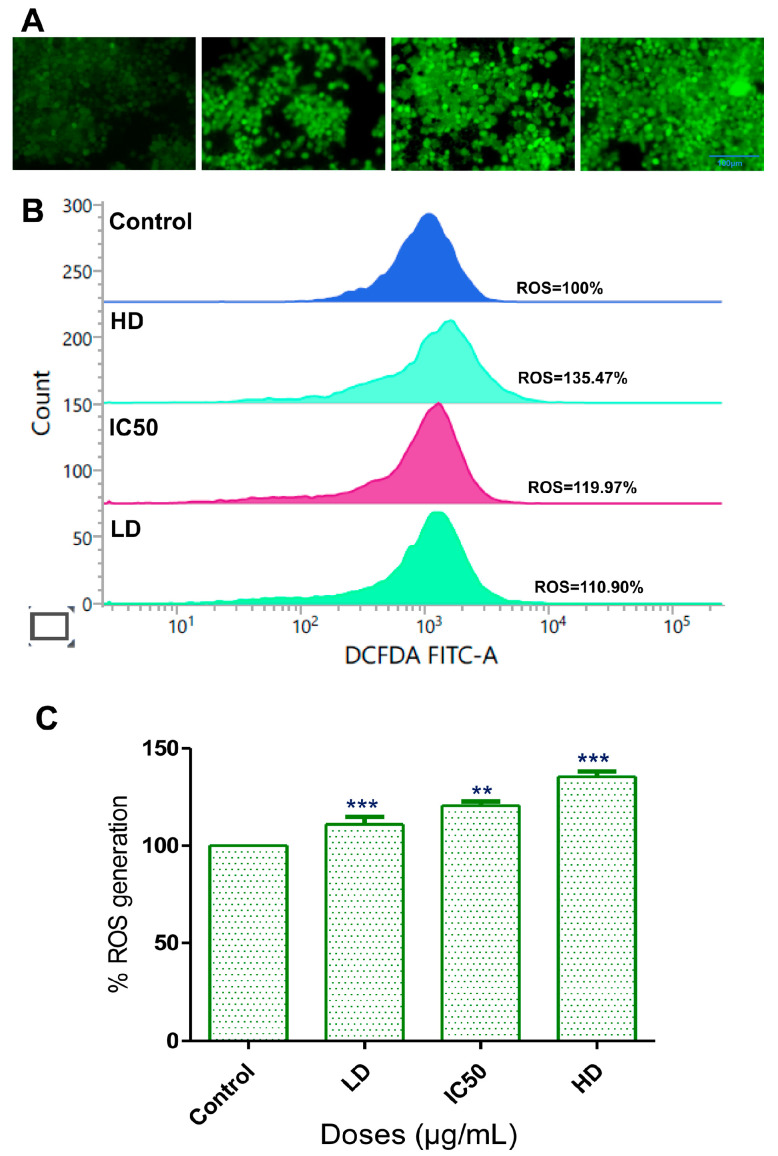
Intracellular ROS generation of MDA-MB-231 cells. (**A**) Images showing intracellular ROS generation induced by *S. persica* bark extract after 12 h incubation under fluorescence microscopy. The fluorescence in the cells is represented as the percentage of ROS production analyzed using flow cytometry. (**B**) The overlay graphs show intracellular ROS generation induced by three effective doses (LD, IC_50_ dose, and HD) of bark extract. (**C**) Graph showing percentage ROS generation as compared to control. ** *p* < 0.01, *** *p* < 0.001 when compared to the control group (n = 3).

Photomicrographic observations showed that, compared with untreated TNBC cells, those exposed to *S. persica* bark extract exhibited a marked increase in ROS intensity (Figure 4A). Flow cytometric quantification further confirmed this finding, indicating that control cells maintained only minimal ROS levels, consistent with normal cellular conditions. In contrast, treatment with bark extract elevated significant ROS production to 10.90%, 16.97%, and 35.47% at LD, IC_50_, and HD, respectively (Figure 4B,C).

### 3.8. MMP Loss Assay by S. persica Bark Extract Against MDA-MB-231 Cells

**Figure 5:**
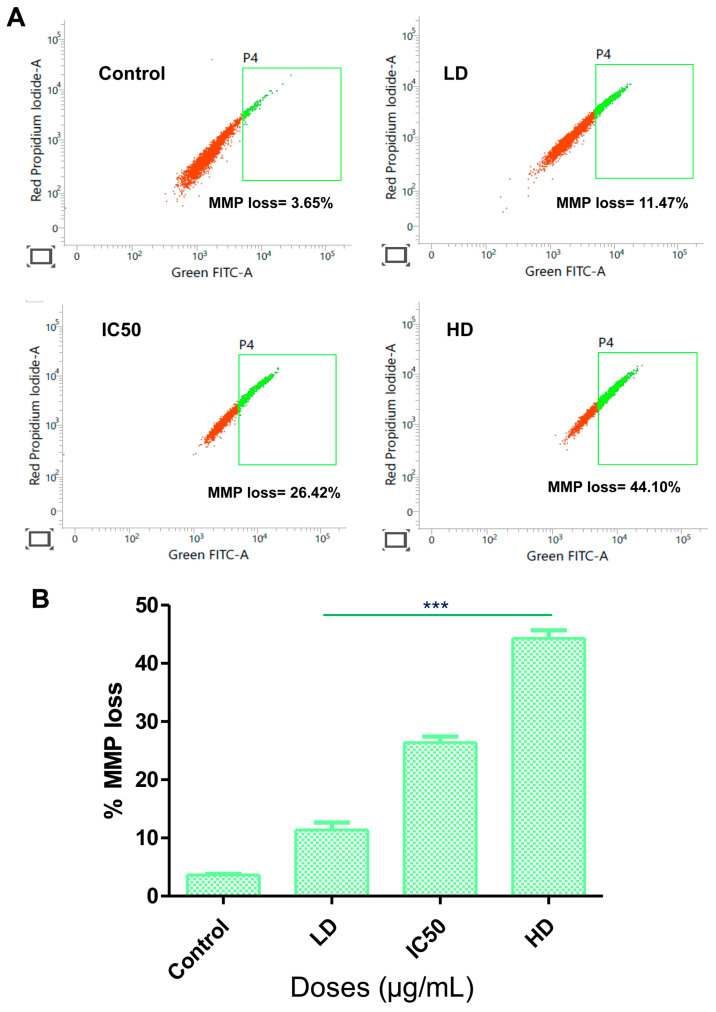
MMP loss of human TNBC MDA-MB-231 cells stained with Rh 123 dye. (**A**) Representative profile of flow-cytometer evaluation exhibits the percentage of cell population with MMP loss. Orange dots represent the total cell population, while cells within the green-gated region (P4) indicate mitochondrial depolarization (MMP loss). (**B**) Graph showing percentage of MMP loss as compared to control. *** *p* < 0.001 when compared to the control group (n = 3).

Flow cytometry results revealed higher MMP loss in treated TNBC MDA-MB-231 cells in contrast to untreated cells. The flow cytometry result for percent MMP loss activity is shown in Figure 5. Findings indicated the decrease in percentage of MMP in treated MDA-MB-231 with increased concentrations of *S. persica* bark extract in a dose-dependent manner. The untreated TNBC cells exhibited 3.65% green fluorescence, while *S. persica* bark extract treatment at LD, IC50 dose, and HD against TNBC cells showed 11.47%, 26.42%, and 44.10% green fluorescence, respectively (Figure 5A,B).

### 3.9. S. persica Bark Extract Induces Cell Cycle Arrest in MDA-MB-231 Cells

**Figure 6:**
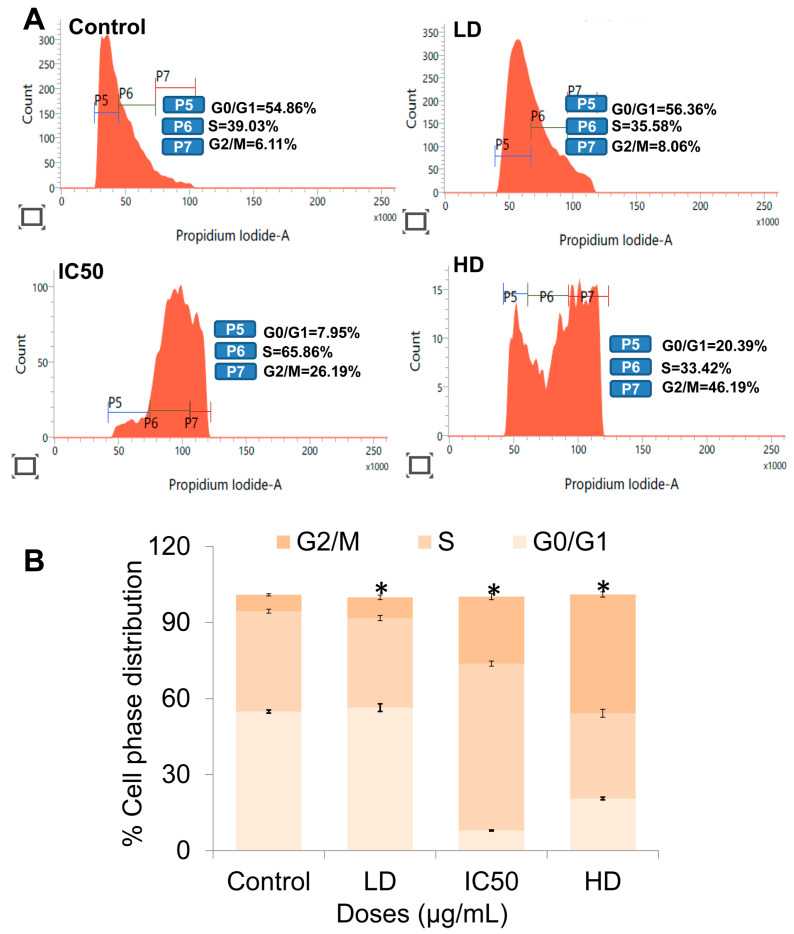
DNA content analysis of MDA-MB-231 cells by flow cytometry. (**A**) Pictorial graph showing the mean proportion of cells in different phases of the cell cycle following treatment with three effective concentrations of *S. persica* bark extract for 24 h. (**B**) Quantification of flow-cytometer data. * *p* < 0.05 when compared to the control group (n = 3).

Cell cycle analysis by flow cytometry was performed against MDA-MB-231 cells after 24 h exposure to *S. persica* bark extract. As depicted in Figure 6A,B, exposure to *S. persica* bark extract greatly increased the proportion of TNBC cells MDA-MB-231 in G2/M phases. There was a potential increase in the proportion of cells in the G2/M phase of the cell cycle after exposure to the extract. These results imply that the G2/M checkpoints of the cell cycle were triggered by bark extract in MDA-MB-231 cells.

### 3.10. Molecular Docking and Interaction Studies

#### 3.10.1. Interaction Analysis of Ligands with EGFR (ErbB1) (PDB: 1IVO)

Rutin showed the strongest binding affinity toward EGFR (−9.8 kcal/mol by Vina; −9.5 kcal/mol by PyRx), outperforming other compounds and suggesting effective competitive inhibition (Table 1). It formed 14 interactions, including seven conventional hydrogen bonds (2.70–3.31 Å) with key amino acid residues such as TYR251, ARG84, ASN86, and ARG231, along with π-donor hydrogen bonds and multiple π–alkyl contacts involving ALA265 and PHE263. Quercetin also demonstrated notable binding (−8.6/−8.4 kcal/mol), interacting through hydrogen bonds with ARG220 and SER205 (2.29–3.07 Å) and π–π T-shaped stacking with HIS209 (4.83–5.46 Å). Chlorogenic acid (−8.2/−7.7 kcal/mol) primarily engaged ARG84 via hydrogen bonding (3.00 Å) and formed hydrophobic π–alkyl interactions with PRO248 and ALA265, indicating accommodation within the ribose-binding pocket. In contrast, fumaric acid exhibited the lowest affinity (−4.3/−4.8 kcal/mol) but maintained three hydrogen bonds (2.93–3.17 Å) with ASN469, ARG470, and GLY471 in the substrate region, suggesting a possible allosteric mode of interaction rather than direct competitive binding (Table S2).

#### 3.10.2. Interaction Analysis of Ligands with FGFR1 (PDB: 1EVT)

Rutin showed the strongest binding to FGFR1, with docking scores of −7.4 kcal/mol (Vina) and −7.5 kcal/mol (PyRx) (Table 1). It formed nine interactions, including five conventional hydrogen bonds (1.96–3.23 Å) with TYR74, GLU82, ASN80, GLN77, and ASP68 within the hinge/activation loop. Additional π–anion and π–π interactions further stabilized the complex, resembling the binding mode of ATP-competitive inhibitors and suggesting effective blockage of the nucleotide-binding site. Chlorogenic acid (−7.0/−6.4 kcal/mol) also showed strong affinity, forming seven hydrogen bonds (2.06–3.25 Å) with residues such as THR96, ASN106, GLY52, GLU87, and GLU90, along with π-based interactions. Quercetin (−7.5/−6.1 kcal/mol) exhibited moderate binding, mainly through hydrophobic and π interactions, indicating accommodation within the receptor’s hydrophobic pocket. Fumaric acid displayed the lowest affinity (−4.3/−4.0 kcal/mol) but maintained a stable hydrogen bond network with LEU111, LYS118, and GLN127 amino acid residues, suggesting a possible allosteric effect rather than direct competition (Table S2). Together, rutin and chlorogenic acid appear to be the most promising FGFR1 inhibitors from *S. persica*, with interaction patterns consistent with potential anti-proliferative activity in FGFR1-driven cancers.

#### 3.10.3. Interaction Analysis of Ligands with FGFR4 (PDB: 4TYE)

Rutin showed the strongest binding to FGFR4, with docking scores of −7.5 kcal/mol (Vina) and −7.9 kcal/mol (PyRx) (Table 1). It formed nine interactions, including five conventional hydrogen bonds (2.76–3.38 Å) with key residues such as ARG566 (bidentate), SER688, THR684, and PRO567, which are important for kinase activation. Additional stabilization was provided by π-donor hydrogen bonding with HIS713 and hydrophobic π interactions with LEU685 and PRO712. Quercetin (−7.5/−7.6 kcal/mol) demonstrated comparable affinity, forming hydrogen bonds with ASP571 (1.86 Å) and HIS713 (2.92 Å), along with π–cation and π–π interactions involving ARG566 and HIS713, and multiple π–alkyl contacts with LEU685 and PRO712. Chlorogenic acid (−6.7/−6.8 kcal/mol) exhibited moderate binding, with interactions including hydrogen bonding to ARG566 and carbon–hydrogen bonds with THR684 and PRO712, supported by π–sigma interaction with LEU685. Fumaric acid (−4.8/−4.5 kcal/mol) showed the lowest affinity but maintained a hydrogen bond network with ARG611, LEU633, and HIS638, indicating possible allosteric binding (Table S2). Thus, rutin and quercetin emerged as the most promising FGFR4 inhibitors from *S. persica*, with key hinge and gatekeeper interactions supporting their potential against FGFR4-associated cancers.

#### 3.10.4. Interaction Analysis of Ligands with Cell Surface GRP78 (csGRP78) (PDB: 3IUC)

Rutin showed the highest binding affinity toward csGRP78 (−9.0 kcal/mol, Vina; −9.3 kcal/mol, PyRx), forming 13 interactions (Table 1). These included four key hydrogen bonds (2.23–2.70 Å) with ASP259, GLU293 (bidentate), and TYR65 within the ATPase nucleotide-binding domain (NBD). Additional stabilization was provided by π–anion interactions (GLU256, GLU310, ASP317), a π–cation interaction with ARG289, and other hydrophobic contacts, indicating strong electrostatic and structural complementarity. Quercetin (−8.0/−7.9 kcal/mol) exhibited stable binding through five hydrogen bonds (2.19–3.26 Å) involving ARG289 (bidentate), SER311, GLU310, and HIS252, along with π–anion interactions and amide–π stacking. Chlorogenic acid (−7.5/−7.3 kcal/mol) formed seven interactions, including five hydrogen bonds (1.96–2.76 Å) with TYR39, THR85, LYS81, and GLU293, supported by additional weak interactions. Fumaric acid (−4.4/−5.0 kcal/mol) displayed comparatively lower affinity but formed multiple hydrogen bonds with residues such as THR37, THR38, TYR39, LYS96, and ASP34, suggesting possible allosteric effects at the substrate-binding region (Table S2). Interactions of rutin and quercetin with csGRP78 further highlight their therapeutic relevance, as this chaperone protein is associated with tumor survival and resistance.

### 3.11. Physicochemical Properties

SwissADME analysis of UHPLC-identified phytoconstituents from *S. persica* bark extract elucidates their drug-like attributes, pivotal for therapeutic viability (Table 2). Rutin (MW 610.52 g/mol; TPSA 269.43 Å^2^) borders Lipinski’s rule-of-five (MW ≤ 500, TPSA ≤ 140 Å^2^ preferred) due to glycosylation, yet its six rotatable bonds and balanced Csp^3^ (0.44) confer flexibility for kinase pocket adaptation, with high HBA/HBD (16/10) enabling extensive hydrogen bonding observed in docking. Elevated TPSA predicts poor CNS penetration but excellent peripheral solubility and target engagement. Quercetin (MW 302.24; TPSA 131.36 Å^2^) exemplifies optimal oral bioavailability (low rotatable bonds:1; LogP~2.0 implied by MR 78.03), and a rigid aromatic scaffold (16 aromatic heavy atoms, 0 Csp^3^). Chlorogenic acid (MW 354.31; TPSA 164.75 Å^2^) maintains drug-likeness with a flexible acyl chain (five rotatable bonds; Csp^3^ 0.38) and phenolic HBA/HBD (9/6), supporting ribose-pocket accommodation despite moderate TPSA. Fumaric acid (MW 116.07; TPSA 74.60 Å^2^) is a quintessential small-molecule lead (minimal heavy atoms: eight; rotatable bonds: two), high rigidity (0 Csp^3^), and low MR (24.41), facilitating allosteric H-bond networks across targets while ensuring rapid absorption and renal clearance. Collectively, these profiles—high aromaticity for π stacking, polar surfaces for solubility, and Lipinski compliance in three of four corroborate docking efficacies and nominate quercetin/rutin for lead optimization in multi-target anticancer regimens (Table S2).

### 3.12. Lipophilicity

Lipophilicity analysis using SwissADME highlights distinct partitioning patterns among *S. persica* phytoconstituents, which are important for ADME optimization (Table S3). Quercetin shows favorable hydrophobicity (consensus LogP 1.23), within the optimal drug-like range (0–3), supporting efficient membrane permeability and interaction with hydrophobic protein pockets. In contrast, rutin is highly hydrophilic (LogP −1.51) due to its glycosylated structure, which enhances aqueous solubility but limits blood–brain barrier penetration. Chlorogenic acid (LogP −0.39) and fumaric acid (LogP −0.35) also display hydrophilic characteristics, suggesting rapid renal clearance and suitability for extracellular targets such as csGRP78, with negative XLOGP3/WLOGP values indicating high water solubility (BCS class I). These differences help explain the docking trends: hydrophobic quercetin favors van der Waals interactions, while more polar compounds like rutin and chlorogenic acid rely on hydrogen bonding, supporting their potential for complementary multi-target strategies in cancer therapy.

### 3.13. Pharmacokinetic Properties

SwissADME analysis indicates favorable ADME characteristics relevant to therapeutic use. Quercetin and fumaric acid show high gastrointestinal absorption, supporting oral administration, whereas rutin exhibits low absorption and P-gp-mediated efflux, suggesting limited bioavailability that may require nanoformulation or parenteral delivery, consistent with its hydrophilic LogP and TPSA values. None of the compounds are predicted to cross the blood–brain barrier, indicating suitability for peripheral targets such as EGFR, FGFR, and csGRP78 rather than CNS applications. Quercetin demonstrates inhibition of multiple CYP isoforms (1A2, 2D6, and 3A4), implying potential drug–drug interactions alongside therapeutic relevance. Skin permeability decreases with increasing polarity (rutin < quercetin), supporting possible topical use. Minimal inhibition of CYP2C19, CYP2C9, and CYP3A4 among the other compounds suggests lower metabolic variability (Table 3).

### 3.14. Drug-likeness Properties

Drug-likeness analysis highlights the therapeutic potential of *S. persica* phytoconstituents (Table S4). Quercetin fully satisfies all five drug-likeness rules with a favorable bioavailability score (0.55). Fumaric acid also shows high bioavailability (0.85) despite minor rule deviations related to its small molecular size, making it suitable for rapid action and clearance. In contrast, rutin exhibits multiple rule violations due to its high molecular weight and polarity, resulting in low bioavailability (0.17), although this may be improved through prodrug approaches or conversion to quercetin. Chlorogenic acid shows limited violations and retains modest bioavailability (0.11). Collectively, compounds with better rule compliance (quercetin, fumaric acid) favor hydrophobic interactions, whereas more polar molecules (rutin, chlorogenic acid) rely on hydrogen bonding, supporting the potential of *S. persica* in polyphenol-based drug development.

### 3.15. Toxicity Potential Analysis

Osiris toxicity predictions indicate generally favorable safety profiles for a potential therapeutic candidate. Rutin and chlorogenic acid show clean toxicological profiles (all green flags), with high drug-likeness scores suggesting a balanced relationship between lipophilicity, solubility, and toxicity (Table 4). Rutin, with the highest score (2.1), supports its long-standing safe use despite minor physicochemical limitations. Quercetin poses potential mutagenic and tumorigenic risks, likely at higher concentrations, although its acceptable irritancy and reproductive profiles, along with its established use at nutraceutical doses, reduce concern. Fumaric acid presents multiple predicted risks, probably due to QSAR overestimation, as existing clinical data (including FDA approval for psoriasis) confirm its safety. Drug-likeness ranking follows rutin > fumaric acid > quercetin > chlorogenic acid. Collectively, the favorable reproductive safety profiles across these compounds support the potential of *S. persica* extract for further oncology research.

## 4. Discussion

Breast cancer is the largest cause of cancer-related mortality among females, affecting around 2.3 million women globally and causing over 670,000 deaths in 2022 [1]. *S. persica* is widely known for its diverse pharmacological properties and has shown cytotoxic effects against several cancer cell lines. The present study was designed to conduct preliminary phytochemical profiling and to evaluate the in vitro anticancer activity of *S. persica* bark extract against TNBC MDA-MB-231 cells, with particular emphasis on its apoptotic mechanisms and safety assessment in Vero cells. In addition, in silico molecular docking was performed to investigate interactions between the identified phytoconstituents and relevant therapeutic membrane receptors. MDA-MB-231 cells are known for their aggressive, mesenchymal-like characteristics and marked drug resistance, which is partly attributed to elevated expression of P-glycoprotein (P-gp) leading to drug efflux, along with increased levels of Nrf2 and associated antioxidant enzymes such as catalase and MnSOD that help counter oxidative stress [17]. The results indicate that *S. persica* bark extract is rich in phenolic and flavonoid compounds and exerts significant cytotoxic effects on TNBC MDA-MB-231 and MDA-MB-468 cell lines. This activity appears to be mediated through ROS-induced mitochondrial apoptosis and cell cycle arrest. Furthermore, molecular docking findings support the potential interaction of these phytochemicals with key therapeutic targets.

Phytochemical evaluation indicated that the *S. persica* bark extract contains substantial amounts of total phenolics and flavonoids, measured at 26.90 ± 0.46 μg GAE/mg and 54.51 ± 0.42 μg QE/mg, respectively. These classes of compounds are well known for their antioxidant and anticancer potential, particularly through their ability to influence key cellular processes such as proliferation, apoptosis, and oxidative stress regulation in cancer cells. Earlier reports have shown that plant-derived polyphenols, including quercetin and rutin, can suppress tumor progression by modulating critical signaling pathways such as PI3K/Akt, MAPK, and NF-κB [18,19]. Accordingly, the elevated levels of phenolics and flavonoids in the *S. persica* bark extract may play a significant role in the anticancer effects observed in this study. UHPLC profiling verified the presence of several biologically active phenolic and flavonoid constituents in the extract, including rutin, quercetin, chlorogenic acid, and fumaric acid. These compounds are well documented for their anticancer potential. For instance, quercetin has been reported to suppress proliferation and trigger apoptosis in breast cancer cells by disrupting mitochondrial function and activating ROS-dependent signaling pathways [20]. In a similar manner, chlorogenic acid demonstrates anti-proliferative activity by modulating oxidative stress and apoptotic processes in breast cancer models [21]. The detection of these phytochemicals in the *S. persica* bark extract strengthens the proposition that its anticancer activity may arise from the coordinated action of multiple molecular mechanisms.

The cytotoxicity assay revealed that *S. persica* bark extract reduced the cell viability of TNBC cells depending upon dose, with an IC_50_ of 144 and 128 µg/mL in MDA-MB-231 and MDA-MB-468 cells, respectively, following 24 h of exposure. Since MDA-MB-231 is widely used in anticancer studies due to its high invasiveness and aggressive tumor behavior, further study was conducted on MDA-MB-231 cells to understand the cell death mechanism. Microscopic examination supported these findings, as treated cells displayed typical apoptotic changes such as shrinkage, rounding, and loss of adherence to the culture surface. In comparison, the extract showed minimal toxicity toward normal Vero cells, suggesting a degree of selectivity for cancer cells. Such selective cytotoxicity is a desirable feature in anticancer agents, as conventional chemotherapeutics frequently damage healthy cells and lead to systemic side effects [22]. Apoptosis represents a key pathway through which anticancer agents eradicate tumor cells. In the present study, DAPI-based nuclear staining demonstrated marked chromatin condensation and nuclear fragmentation in bark extract-treated cells, confirming the occurrence of apoptotic cell death. These observations were supported by Annexin V-FITC/PI flow cytometry analysis, which showed a concentration-dependent increase in apoptotic and late apoptotic/secondary necrotic cell populations following treatment with *S. persica* bark extract. Lower treatment concentrations predominantly induced apoptotic features, whereas higher concentrations near or above the IC_50_ likely involved mixed cell death responses associated with severe oxidative and mitochondrial damage. Induction of apoptosis is particularly advantageous in cancer therapy, as it enables the selective removal of malignant cells while minimizing inflammation and necrotic damage [23].

A major observation of the present study is the involvement of oxidative stress in bark extract-induced cytotoxicity. Flow cytometric analysis revealed a significant, dose-dependent rise in intracellular reactive oxygen species (ROS) following treatment with the extract. ROS are known to exert a dual influence in cancer: while controlled levels support tumor growth and survival, excessive accumulation leads to oxidative damage and initiates apoptotic cell death [24]. Several phytochemicals are reported to exert anticancer effects by elevating ROS beyond the tolerable limit of cancer cells, thereby promoting mitochondrial dysfunction and activating apoptotic pathways [25]. In line with this mechanism, bark extract caused a marked reduction in MMP in MDA-MB-231 cells. Mitochondria are central to the intrinsic apoptotic pathway, and disruption of MMP facilitates the release of pro-apoptotic factors such as cytochrome c, which in turn activates the caspase cascade leading to programmed cell death. The observed dose-dependent loss of MMP therefore indicates that bark extract induces apoptosis via a mitochondrial-mediated pathway. Furthermore, bark extract significantly arrested the cell cycle at the G_2_/M phase. Cell cycle progression is governed by cyclins, cyclin-dependent kinases, and checkpoint regulators that ensure proper DNA replication and division. Interference with these regulatory systems can suppress cell proliferation and promote apoptosis [26]. The accumulation of cells in the G_2_/M phase suggests that bark extract disrupts normal cell cycle progression, thereby inhibiting the growth of TNBC cells.

The combined molecular docking and ADME analyses indicate that *S. persica* bark-derived phytoconstituents possess notable multi-target anticancer potential, particularly rutin, quercetin, and chlorogenic acid. Among these, rutin exhibited the strongest binding affinities across key oncogenic targets, including EGFR, FGFR1/4, and csGRP78, suggesting its ability to act as a broad-spectrum inhibitor of cancer-related signaling pathways. Its interaction with EGFR, characterized by multiple hydrogen bonds and π interactions, supports a possible ATP-competitive inhibitory mechanism similar to established tyrosine kinase inhibitors. Rutin and quercetin also showed stable binding with FGFR1 and FGFR4, targeting critical residues involved in kinase activation, which may contribute to the suppression of tumor growth and resistance mechanisms. Chlorogenic acid demonstrated comparatively moderate but stable interactions, indicating a supportive modulatory role. GRP78 is known to function as a protective chaperone that supports TNBC cell survival under cellular stress conditions and contributes to chemoresistance by suppressing apoptosis, enhancing drug efflux, and promoting immune evasion, thereby facilitating tumor progression and recurrence [27,28]. Therefore, csGRP78 has emerged as an attractive therapeutic target for overcoming resistance in TNBC. In the present docking analysis, rutin exhibited the strongest binding affinity toward csGRP78 with 13 molecular interactions, and this strong interaction profile suggests its potential role in modulating GRP78-associated survival signaling, supporting its possible contribution to the anticancer activity of *S. persica* bark extract. ADME analysis identified quercetin as the most promising candidate due to its favorable drug-likeness and absorption profile. In contrast, rutin showed limited bioavailability, suggesting the need for formulation strategies to enhance its therapeutic utility. Chlorogenic and fumaric acids demonstrated good safety and solubility, supporting their possible synergistic roles.

Although flavonoids such as quercetin and rutin are recognized as redox-active compounds with potential PAINS-associated behavior under certain assay conditions, the anticancer activity observed in this study was supported by multiple independent cellular assays, including apoptosis analysis, ROS generation, mitochondrial membrane depolarization, and cell cycle arrest. The selective cytotoxicity of *S. persica* bark extract toward breast cancer cells, with comparatively lower toxicity in Vero cells, further suggests that the observed effects are not solely due to nonspecific assay interference. Previous studies have also demonstrated that quercetin and rutin regulate defined molecular pathways associated with ROS-mediated apoptosis and cell cycle disruption in different cancers, such as oral cancer KON and cervical cancer Caski cell lines [29,30].

Vero cells were used in the present study as a preliminary non-cancerous epithelial model to assess general cytotoxic safety; however, future studies using normal human mammary epithelial cells such as MCF-10A would provide a more physiologically relevant evaluation of breast cancer selectivity. In addition, inclusion of standard chemotherapeutic agents such as doxorubicin or cisplatin as positive controls would allow direct comparison of therapeutic efficacy. The relatively moderate IC_50_ values obtained for the crude extract indicate the need for bioactivity-guided fractionation and isolation of active phytoconstituents to improve potency. Moreover, the study did not include caspase activation assays or in vivo validation, which will be important in future investigations to confirm apoptotic pathways, pharmacokinetics, systemic toxicity, and therapeutic efficacy under physiological conditions.

## 5. Conclusions

In conclusion, the present study provides experimental and computational evidence supporting the anticancer potential and multi-targeted activity of phytochemicals from *S. persica* bark extract against TNBC cells, which provides a foundation for future studies to elucidate their therapeutic applications. The observed biological activities may be attributed to the presence of bioactive phenolic and flavonoid compounds such as quercetin, rutin, and chlorogenic acid. Collectively, these findings support the traditional use of *S. persica* and identify its polyphenols as promising multi-target anticancer agents, although further experimental validation is necessary to confirm their potential therapeutic candidate.

## Figures and Tables

**Table 1:** Binding energy and interaction studies of identified phytomolecules from *S. persica* bark extract against therapeutic membrane receptors of TNBC, *viz*. Epidermal growth factor receptor (EGFR/ErbB1), fibroblast growth factor receptors (FGFR1), fibroblast growth factor receptors (FGFR4), and cell surface GRP78 (csGRP78) through two docking tools, Autodock Vina and PyRx. 2D and 3D Interactions were visualized using BIOVIA Discovery Studio.

Compounds Name	A. Vina BA (kcal/mol)	PyRx BA (kcal/mol)	3-D Interaction	2-D Interaction
Interaction analysis of Epidermal growth factor receptor (EGFR/ErbB1) (PDB ID: 1IVO)				
Rutin	−9.8	−9.5	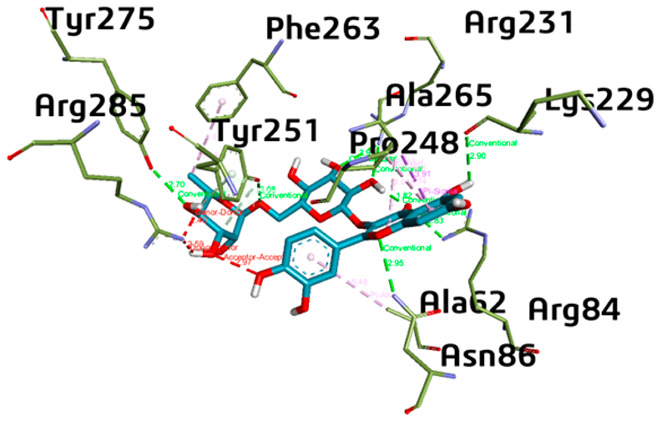	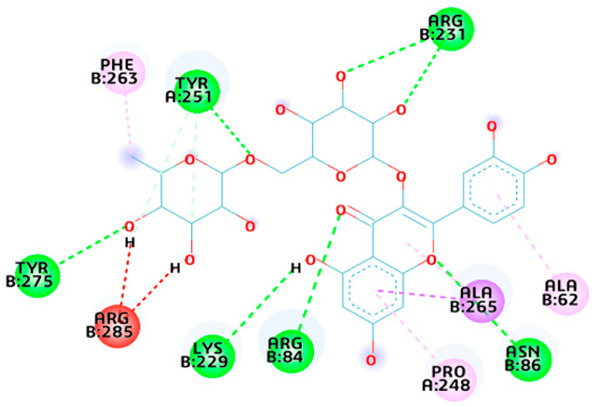
Quercetin	−8.6	−8.4	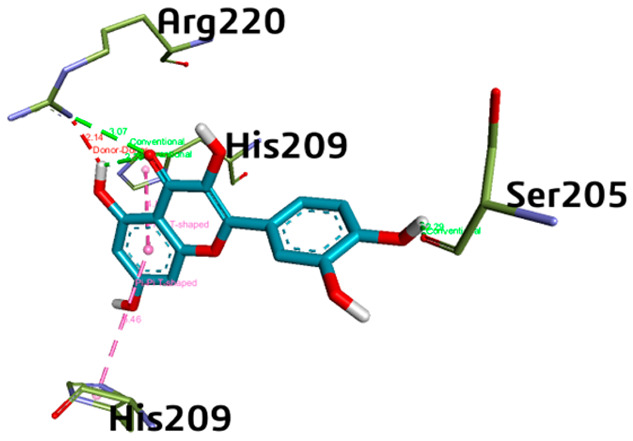	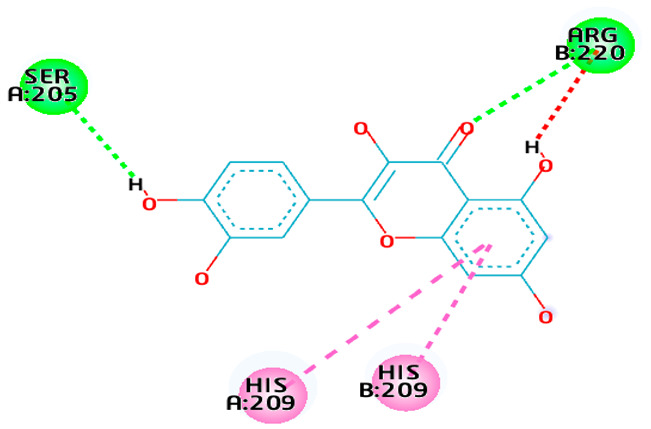
Chlorogenic acid (1794427)	−8.2	−7.7	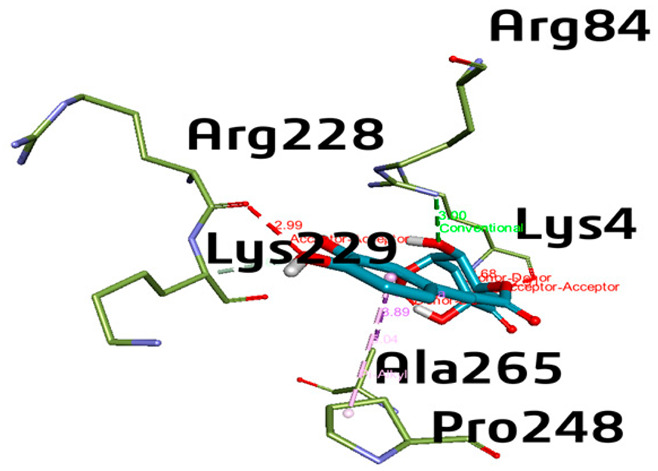	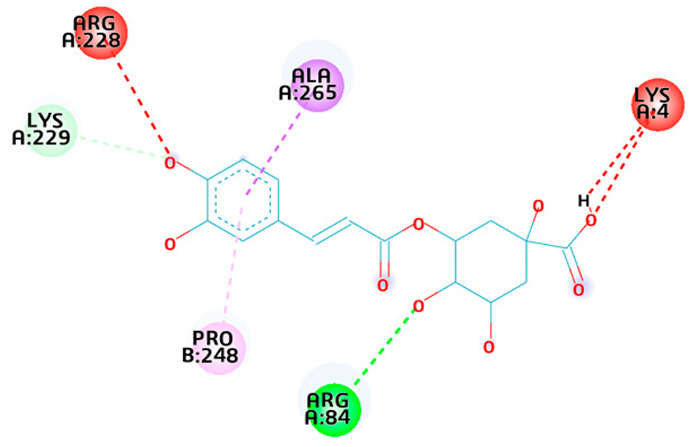
Fumaric acid	−4.3	−4.8	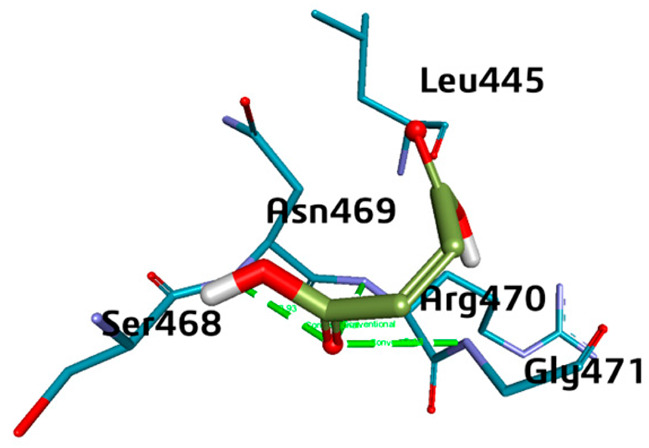	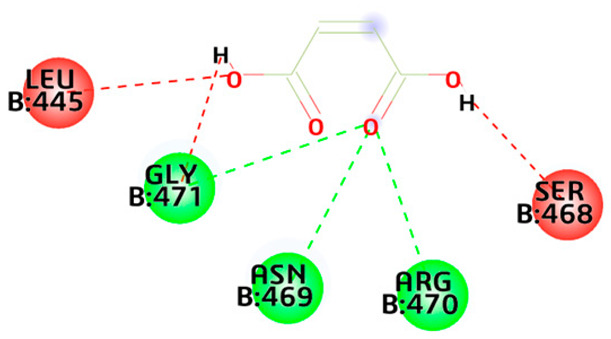
Interaction analysis of Fibroblast growth factor receptors (FGFR1) (PDB ID: 1EVT)				
Rutin	−7.4	−7.5	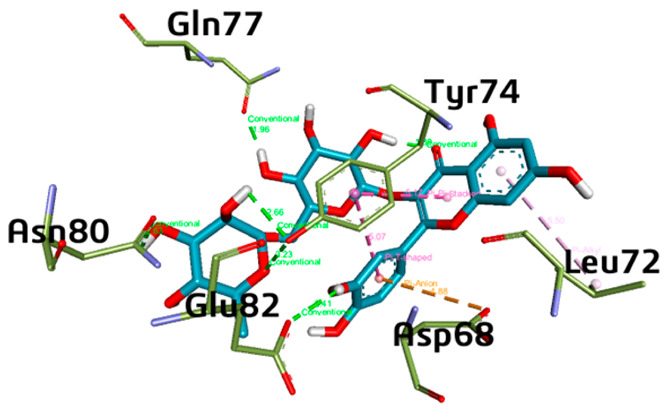	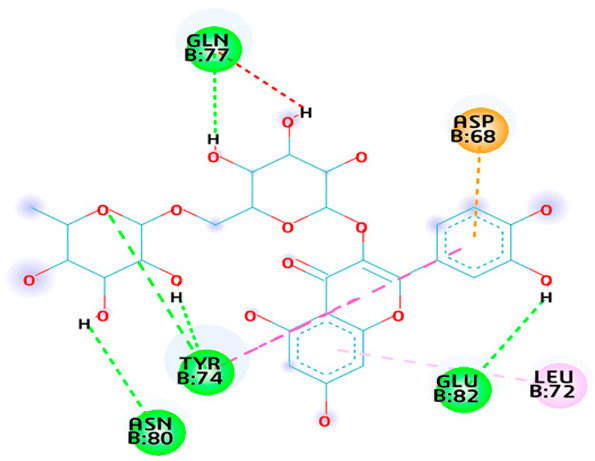
Chlorogenic acid	−7.0	−6.4	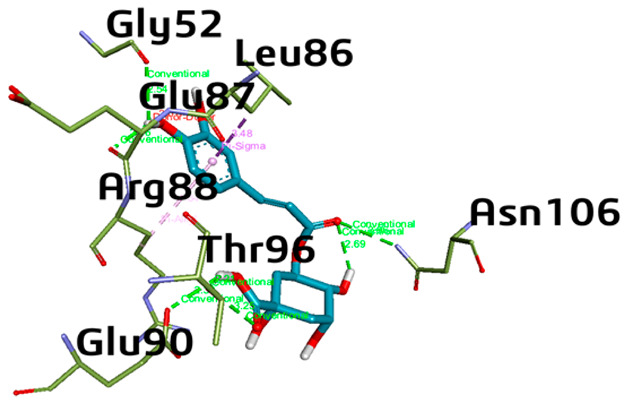	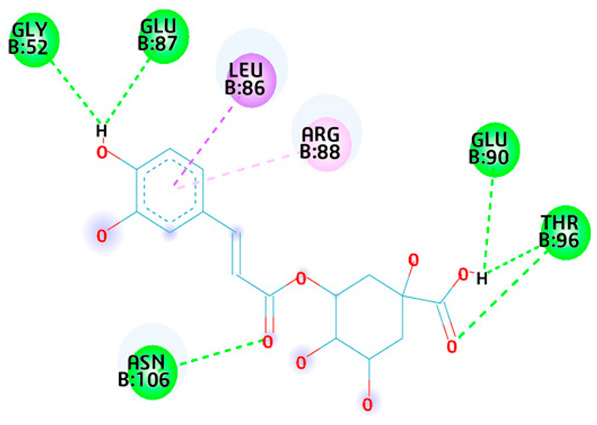
Quercetin	−7.5	−6.1	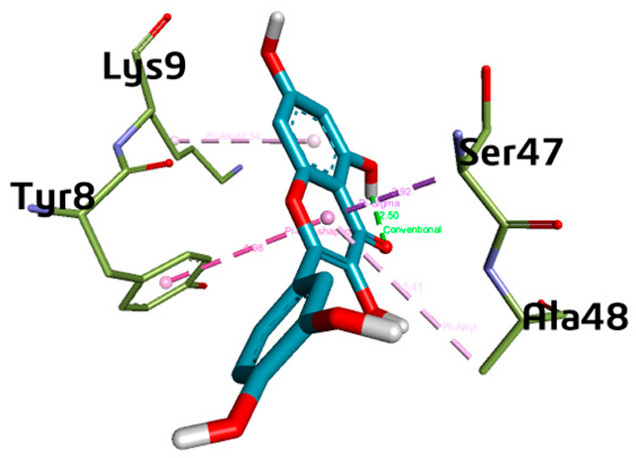	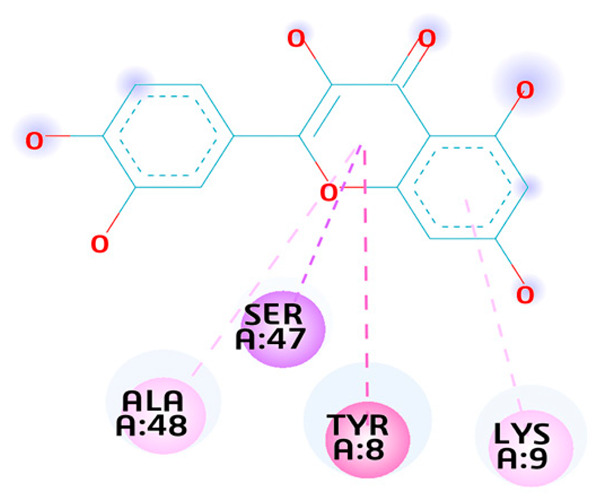
Fumaric acid	−4.3	−4	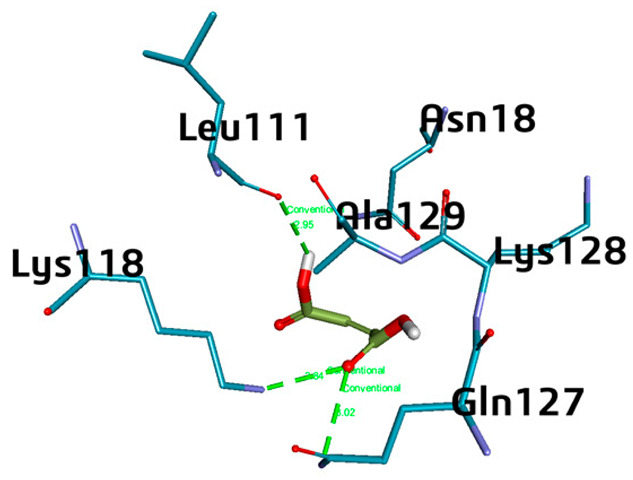	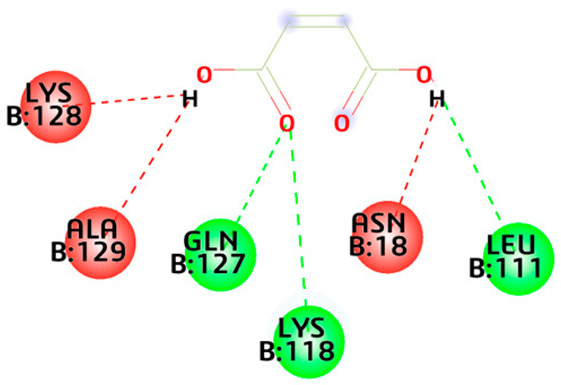
Interaction analysis of Fibroblast growth factor receptors (FGFR4) (PDB ID: 4TYE)				
Rutin	−7.5	−7.9	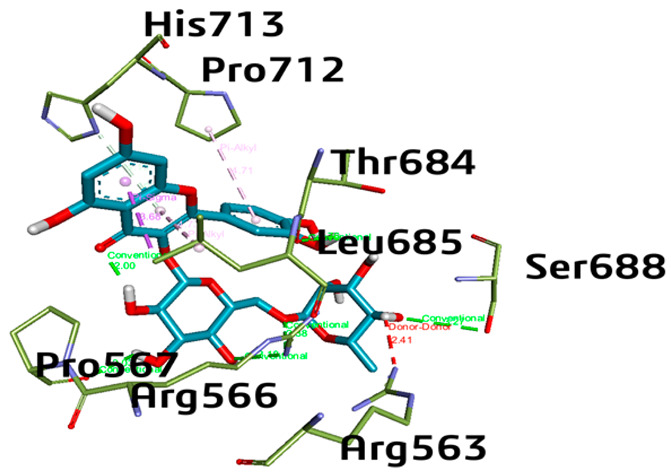	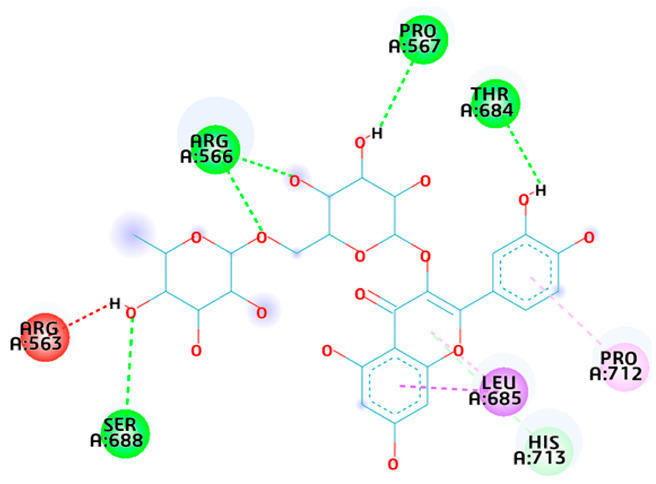
Quercetin	−7.5	−7.6	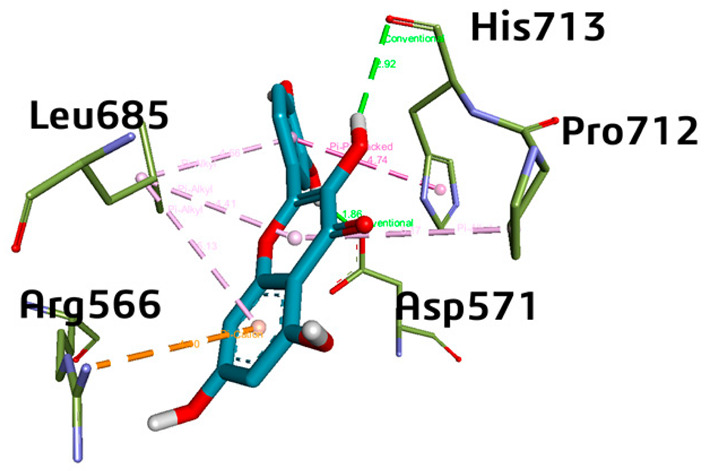	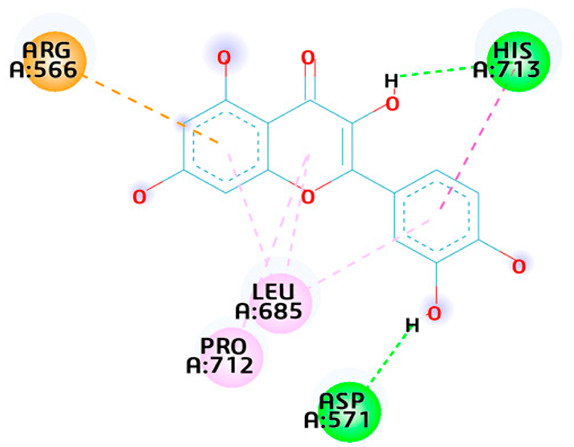
Chlorogenic acid	−6.7	−6.8	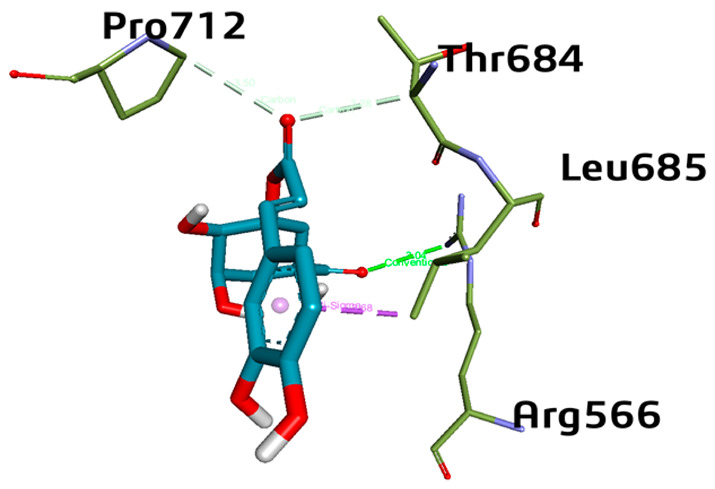	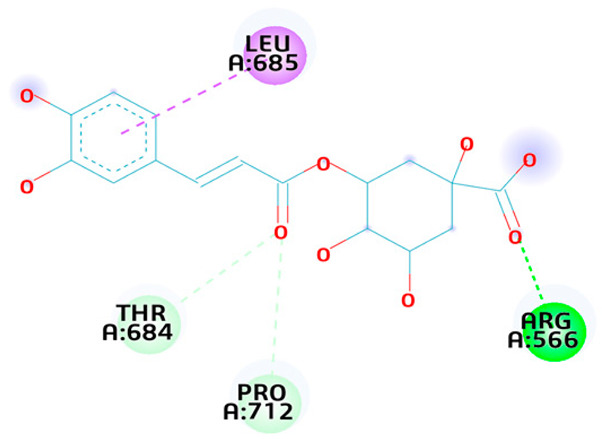
Fumaric acid	−4.8	−4.5	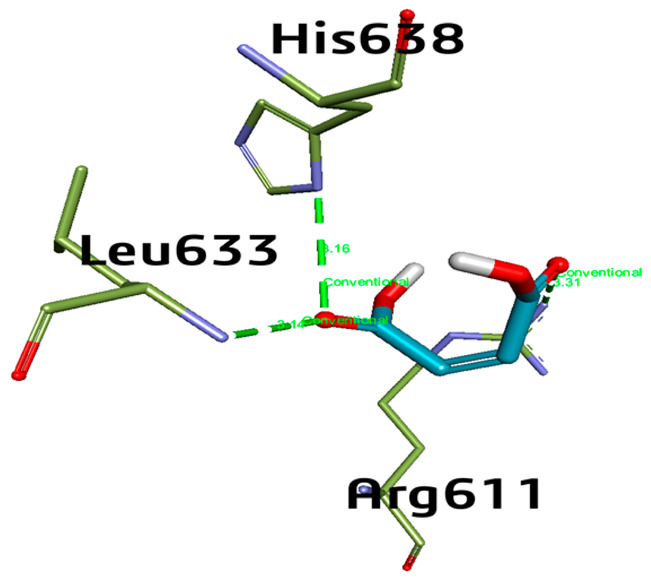	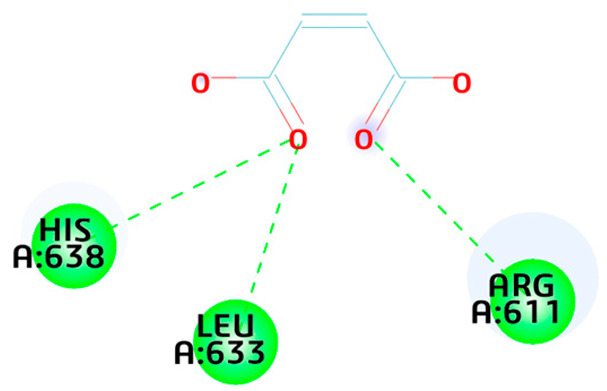
Interaction analysis of Cell Surface GRP78 (csGRP78) (PDB ID: 3IUC)				
Rutin	−9.0	−9.3	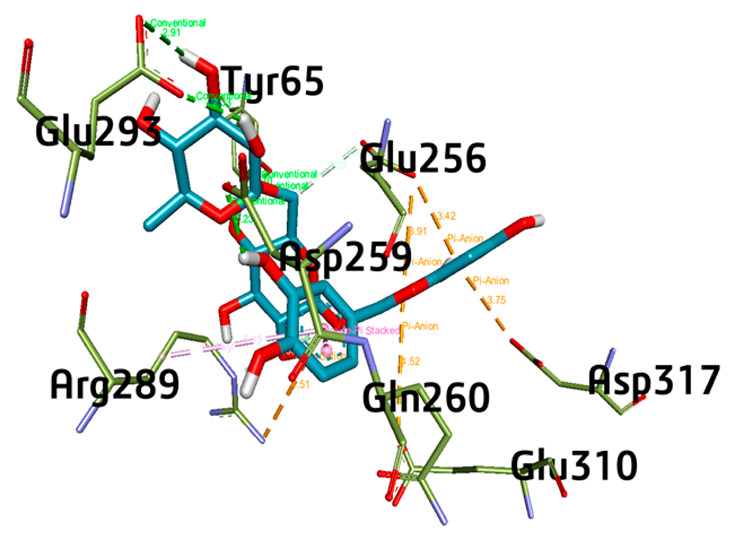	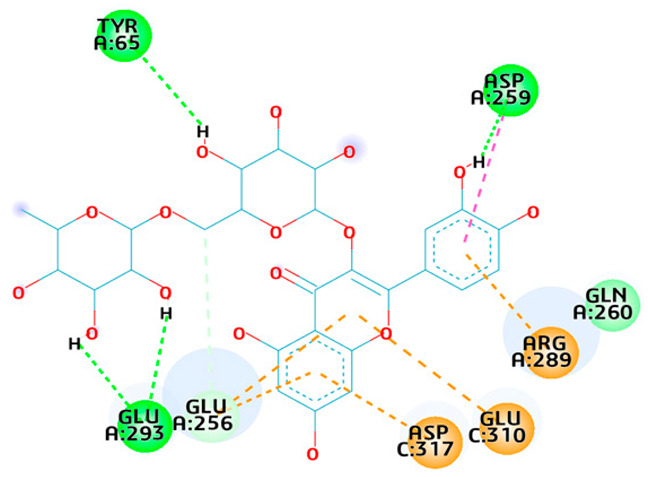
Quercetin	−8.0	−7.9	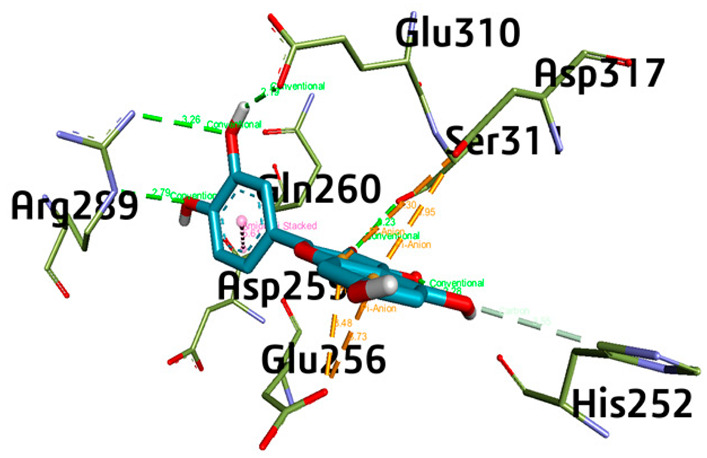	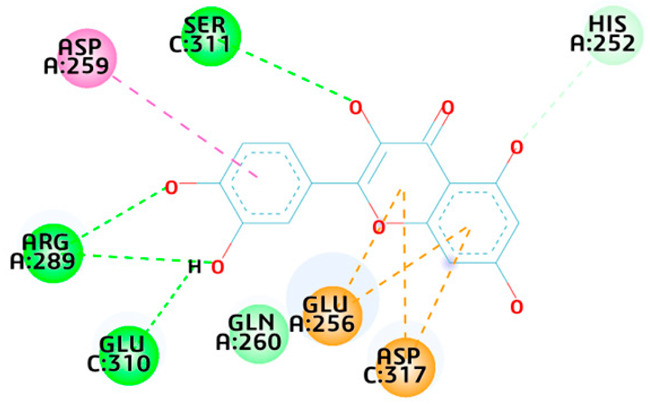
Chlorogenic acid	−7.5	−7.3	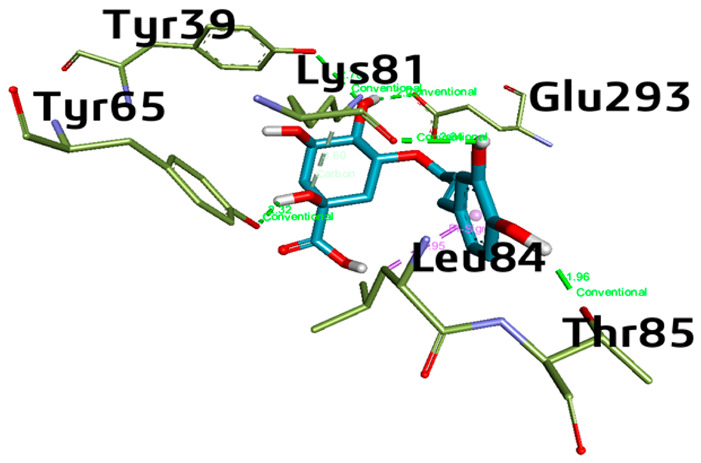	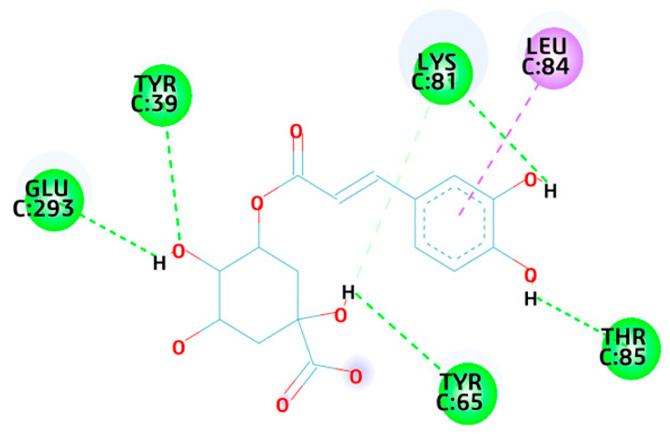
Fumaric acid	−4.4	−5	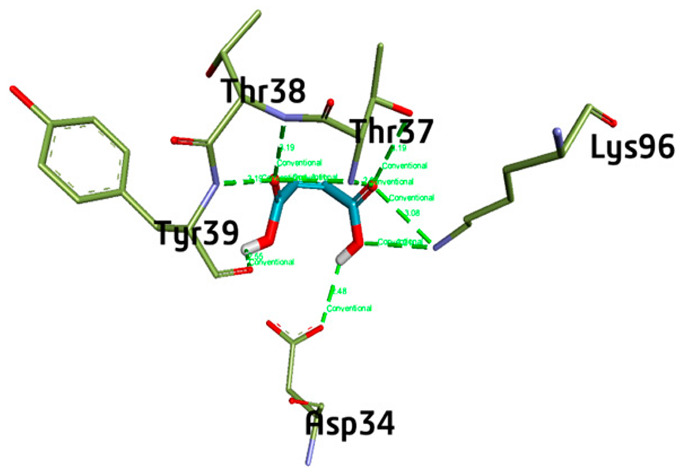	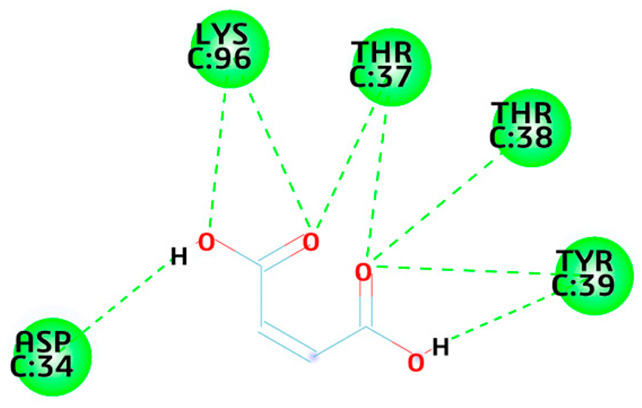

**Table 2:** Physicochemical properties of *S. persica* bark phytoconstituents obtained through UHPLC analysis.

S. No.	Phytoconstituents	Formula	MW	Heavy Atoms	Aromatic Heavy Atoms	Fraction Csp3	Rotatable Bonds	H-Bond Acceptors	H-Bond Donors	MR	TPSA
1.	Rutin	C_27_H_30_O_16_	610.52	43	16	0.44	6	16	10	141.38	269.43
2.	Quercetin	C_15_H_10_O_7_	302.24	22	16	0	1	7	5	78.03	131.36
3.	Chlorogenic acid	C_16_H_18_O_9_	354.31	25	6	0.38	5	9	6	83.5	164.75
4.	Fumaric acid	C_4_H_4_O_4_	116.07	8	0	0	2	4	2	24.41	74.6

**Table 3:** Pharmacokinetic properties of *S. persica* bark phytoconstituents obtained through UHPLC.

S. No.	Phytoconstituents	GI Absorption	BBB Permeant	Pgp Substrate	CYP1A2 Inhibitor	CYP2C19 Inhibitor	CYP2C9 Inhibitor	CYP2D6 Inhibitor	CYP3A4 Inhibitor	log Kp (cm/s)
1.	Rutin	Low	No	Yes	No	No	No	No	No	−10.26
2.	Quercetin	High	No	No	Yes	No	No	Yes	Yes	−7.05
3.	Chlorogenic acid	Low	No	No	No	No	No	No	No	−8.76
4.	Fumaric acid	High	No	No	No	No	No	No	No	−7.25

**Table 4:** Toxicity potential studies of *S. persica* bark phytoconstituents obtained through UHPLC.

S. No.	Phytoconstituents	Mutagenic	Tumorigenic	Irritant	Reproductive Effect	Drug Likeness
1.	Rutin	Green	Green	Green	Green	2.1
2.	Quercetin	Red	Green	Green	Green	1.6
3.	Chlorogenic acid	Green	Green	Green	Green	0.17
4.	Fumaric acid	Green	Green	Red	Green	1.9

## Data Availability

The original contributions presented in the study are included in the article/Supplementary Material; further inquiries can be directed to the corresponding authors.

## Supplementary Materials

The following supporting information can be downloaded at: https://www.mdpi.com/article/10.3390/life16060943/s1, Figure S1: Gallic Acid Standard Graph; Figure S2: Quercetin Standard Graph; Figure S3: Cytotoxic test of *S. persica* bark extract on human TNBC breast cancer MDA-MB-468 cells; Table S1: Docking Grid Parameters for Target Proteins; Table S2: Binding Interaction studies of identified phytomolecules from *S. persica* bark extract against therapeutic membrane receptors of TNBC, *viz.* Epidermal Growth Factor Receptor (EGFR/ErbB1), Fibroblast growth factor receptors (FGFR1), Fibroblast growth factor receptors (FGFR4), and Cell Surface GRP78 (csGRP78); Table S3: Lipophilicity of *S. persica* bark phytoconstituents identified through UHPLC analysis; Table S4: Drug likeness properties of *S. persica* phytoconstituents identified through UHPLC.

## Abbreviations

The following abbreviations are used in this manuscript:

TNBC	triple-negative breast cancer
UHPLC	Ultra-High Performance Liquid Chromatography
TFC	total flavonoid contents
TPC	total phenolic contents
MTT	3-(4,5-dimethylthiazol-2-yl)-2,5-diphenyltetrazolium bromide
ROS	reactive oxygen species
MMP	mitochondrial membrane potential
EGFR	epidermal growth factor receptor
FGFR	fibroblast growth factor receptor
csGRP78	cell surface glucose-regulated protein 78
TRPC3	Transient Receptor Potential Canonical 3
GLOBOCAN	Global Cancer Observatory
ER	estrogen receptor
PR	progesterone receptor
HER2	human epidermal growth factor receptor-2
FBS	fetal bovine serum
DCFH-DA	2′,7′-dichlorodihydrofluorescein diacetate
Rh123	Rhodamine 123
DMSO	Dimethyl Sulfoxide
LD	low dose
HD	high dose
IC50	Half-Maximal Inhibitory Concentration
