# Supplementary material for: Bioactive Profiling and Evaluation of Anti-Proliferative Potential of Salvadora persica Bark Extract in Triple-Negative Breast Cancer Cells: An In Vitro and Computational Analysis
Source: Life (Basel). 2026 Jun 3;16(6):943. doi: 10.3390/life16060943 (PMC13302017; doi:10.3390/life16060943)
Supplement: Supplementary file 1 [file life-16-00943-s001.zip › life-4324520-supplementary.pdf]

Supplementary Material

Supplementary Table

Table S1. Docking Grid Parameters for Target Proteins.

S. No.	Target Protein	Center (x, y, z) Å
1.	EGFR (1IVO)	79.412000, 66.806000, 46.721000
2.	FGFR1 (1EVT)	16.832000, 39.993000, 38.906000
3.	FGFR4 (4TYE)	-4.737000, 22.0044000, 5.836000
4.	csGRP78 (3IUC)	-14.431000, -3.842000, 36.975000

Table S2. Binding Interaction studies of identified phytomolecules from *S. persica* bark extract against therapeutic membrane receptors of TNBC, *viz.* Epidermal Growth Factor Receptor (EGFR / ErbB1), Fibroblast growth factor receptors (FGFR1), Fibroblast growth factor receptors (FGFR4), and Cell Surface GRP78 (csGRP78).

S. No.	Compounds Name	Interacting amino acid residues	Bond-Distance (Å)	Bond-Types
**Interaction analysis of Epidermal Growth Factor Receptor (EGFR / ErbB1) (PDB ID: 1IVO)**				
1.	Rutin	TYR251	3.07879	Conventional
		ARG84	2.82624	Hydrogen Bond
		ASN86	2.95234	Conventional
		ARG231	3.3118	Hydrogen Bond
		ARG231	2.9116	Conventional
		TYR275	2.70437	Hydrogen Bond
		LYS229	2.90118	Conventional
		TYR251	3.71361	Hydrogen Bond
		TYR251	3.30488	Conventional
		ALA265	3.91352	Hydrogen Bond
		PHE263	4.81908	Conventional
		ALA265	4.01428	Hydrogen Bond

		PRO248	4.73738	Conventional
		ALA62	5.47675	Hydrogen Bond
				Carbon Hydrogen
				Bond
				Pi-Donor Hydrogen
				Bond
				Pi-Sigma
				Pi-Alkyl
				Pi-Alkyl
				Pi-Alkyl
				Pi-Alkyl
2.	Quercetin	ARG220	3.0727	Conventional
		SER205	2.28802	Hydrogen Bond
		HIS209	5.4639	Conventional
		HIS209	4.82575	Hydrogen Bond
				Pi-Pi T-shaped
				Pi-Pi T-shaped
3.	Chlorogenic acid (1794427)	ARG84	2.9982	Conventional
		LYS229	3.59912	Hydrogen Bond
		ALA265	3.88546	Carbon Hydrogen
		PRO248	5.04481	Bond
				Pi-Sigma
				Pi-Alkyl
4.	Fumaric acid	ASN469	2.92843	Hydrogen Bond
		ARG470	3.10533	Hydrogen Bond
		GLY471	3.16945	Hydrogen Bond
	**Interaction analysis of Fibroblast growth factor receptors (FGFR1) (PDB ID: 1EVT)**			
1.	Rutin	TYR74	3.23331	Conventional
		GLU82	2.4115	Hydrogen Bond
		TYR74	2.66105	Conventional
		ASN80	2.63179	Hydrogen Bond
		GLN77	1.95558	Conventional
		ASP68	4.88314	Hydrogen Bond
		TYR74	5.17339	Conventional

		TYR74	5.06984	Hydrogen Bond
		LEU72	5.49706	Conventional
				Hydrogen Bond
				Pi-Anion
				Pi-Pi Stacked
				Pi-Pi T-shaped
				Pi-Alkyl
2.	Chlorogenic acid	THR96	3.24512	Conventional
		ASN106	2.96034	Hydrogen Bond
		GLY52	2.53741	Conventional
		GLU87	2.05641	Hydrogen Bond
		GLU90	2.39396	Conventional
		THR96	2.21156	Hydrogen Bond
		LEU86	3.48351	Conventional
		ARG88	4.84048	Hydrogen Bond
				Conventional
				Hydrogen Bond
				Conventional
				Hydrogen Bond
				Pi-Sigma
				Pi-Alkyl
3.	Quercetin	SER47	3.92328	Pi-Sigma
		TYR8	4.97502	Pi-Pi T-shaped
		ALA48	5.40857	Pi-Alkyl
		LYS9	4.54115	Pi-Alkyl
4.	Fumaric acid	LEU111	2.9527	Conventional
		LYS118	2.83784	Hydrogen Bond
		GLN127	3.01647	Conventional
				Hydrogen Bond
				Conventional
				Hydrogen Bond
	**Interaction analysis of Fibroblast growth factor receptors (FGFR4) (PDB ID: 4TYE)**			
1.	Rutin	ARG566	3.3841	Conventional
		ARG566	3.18357	Hydrogen Bond

		SER688	3.2656	Conventional
		THR684	2.76274	Hydrogen Bond
		PRO567	3.03041	Conventional
		HIS713	4.18571	Hydrogen Bond
		LEU685	3.68257	Conventional
		LEU685	4.73008	Hydrogen Bond
		PRO712	4.7091	Conventional
				Hydrogen Bond
				Pi-Donor Hydrogen
				Bond
				Pi-Sigma
				Pi-Alkyl
				Pi-Alkyl
2.	Quercetin	ASP571	1.85662	Conventional
		HIS713	2.9167	Hydrogen Bond
		ARG566	4.40053	Conventional
		HIS713	4.74411	Hydrogen Bond
		LEU685	4.40636	Pi-Cation
		PRO712	5.17284	Pi-Pi Stacked
		LEU685	5.12815	Pi-Alkyl
		LEU685	4.66376	Pi-Alkyl
				Pi-Alkyl
				Pi-Alkyl
3.	Chlorogenic acid	ARG566	3.03887	Conventional
		THR684	3.28452	Hydrogen Bond
		PRO712	3.50289	Carbon Hydrogen
		LEU685	3.68021	Bond
				Carbon Hydrogen
				Bond
				Pi-Sigma
4.	Fumaric acid	ARG611	3.31136	Conventional
		LEU633	3.14239	Hydrogen Bond
		HIS638	3.15774	Conventional
				Hydrogen Bond

				Conventional
				Hydrogen Bond
**Interaction analysis of Cell Surface GRP78 (csGRP78) (PDB ID: 3IUC)**				
1.	Rutin	ASP259	2.22576	Conventional
		GLU293	2.32919	Hydrogen Bond
		GLU293	2.91032	Conventional
		TYR65	2.69597	Hydrogen Bond
		GLU256	3.19768	Conventional
		ARG289	4.50649	Hydrogen Bond
		GLU256	3.9119	Conventional
		GLU256	3.42207	Hydrogen Bond
		GLU310	4.52348	Carbon Hydrogen
		ASP317	3.75122	Bond
		ARG289	3.59864	Pi-Cation
		ASP259	4.96503	Pi-Anion
		ARG289	5.15336	Pi-Anion
				Pi-Anion
				Pi-Anion
				Pi-Donor Hydrogen
				Bond
				Amide-Pi Stacked
				Pi-Alkyl
2.	Quercetin	ARG289	2.79157	Conventional
		ARG289	3.25939	Hydrogen Bond
		SER311	3.23491	Conventional
		GLU310	2.19229	Hydrogen Bond
		HIS252	2.27929	Conventional
		GLU256	3.55011	Hydrogen Bond
		GLU256	3.48408	Conventional
		ASP317	3.73144	Hydrogen Bond
		ASP317	4.30479	Conventional
		ASP259	3.94662	Hydrogen Bond
		GLN260	5.60701	Carbon Hydrogen
				Bond

				Pi-Anion
				Pi-Anion
				Pi-Anion
				Pi-Anion
				Amide-Pi Stacked
3.	Chlorogenic acid	TYR39	2.76287	Conventional
		THR85	1.95954	Hydrogen Bond
		LYS81	2.33646	Conventional
		GLU293	2.22365	Hydrogen Bond
		TYR65	2.32331	Conventional
		LYS81	3.59542	Hydrogen Bond
		LEU84	3.94603	Conventional
				Hydrogen Bond
				Conventional
				Hydrogen Bond
				Carbon Hydrogen
				Bond
				Pi-Sigma
4.	Fumaric acid	THR37	2.95621	Conventional
		THR37	3.11246	Hydrogen Bond
		THR37	3.18675	Conventional
		THR38	3.1925	Hydrogen Bond
		TYR39	3.19448	Conventional
		LYS96	3.08479	Hydrogen Bond
		LYS96	3.20569	Conventional
		ASP34	2.47708	Hydrogen Bond
		TYR39	2.54531	Conventional
				Hydrogen Bond
				Conventional
				Hydrogen Bond
				Conventional
				Hydrogen Bond
				Conventional
				Hydrogen Bond

Conventional Hydrogen Bond

Table S3. Lipophilicity of *S. persica* bark phytoconstituents identified through UHPLC analysis.

S. No.	Phytoconstituents	iLOGP	XLOGP3	WLOGP	MLOGP	Silicos-IT Log P	Consensus Log P
1.	Rutin	0.46	-0.33	-1.69	-3.89	-2.11	-1.51
2.	Quercetin	1.63	1.54	1.99	-0.56	1.54	1.23
3.	Chlorogenic acid	0.87	-0.42	-0.75	-1.05	-0.61	-0.39
4.	Fumaric acid	0.32	-0.34	-0.29	-0.64	-0.81	-0.35

Table S4. Drug likeness properties of *S. persica* phytoconstituents identified through UHPLC.

S. No.	Phytoconstituents	Lipinski violations	Ghose violations	Veber violations	Egan violations	Muegge violations	Bioavailability Score
1.	Rutin	3	4	1	1	4	0.17
2.	Quercetin	0	0	0	0	0	0.55
3.	Chlorogenic acid	1	1	1	1	2	0.11
4.	Fumaric acid	0	3	0	0	2	0.85

Supplementary Figures

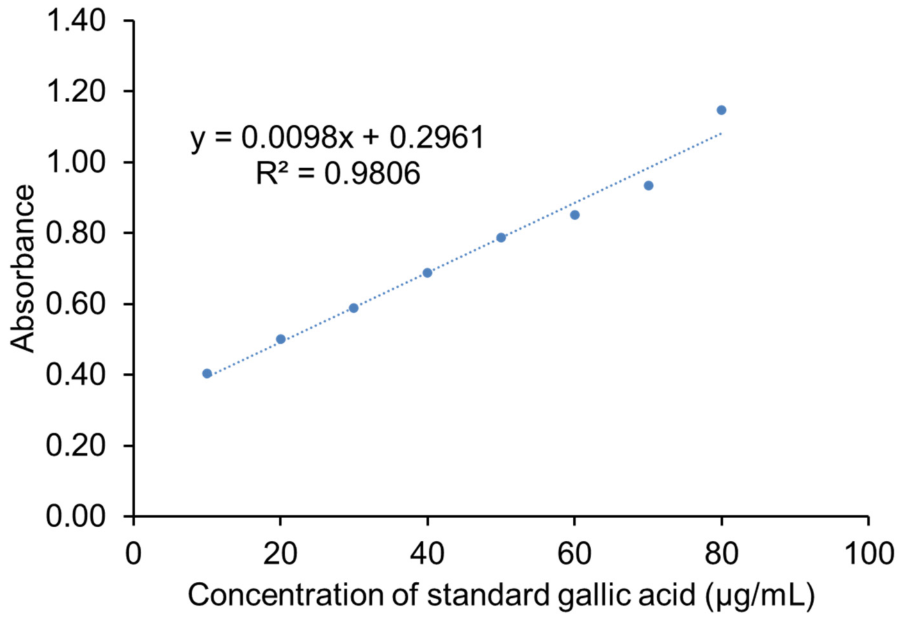

Figure S1: Gallic Acid Standard Graph.

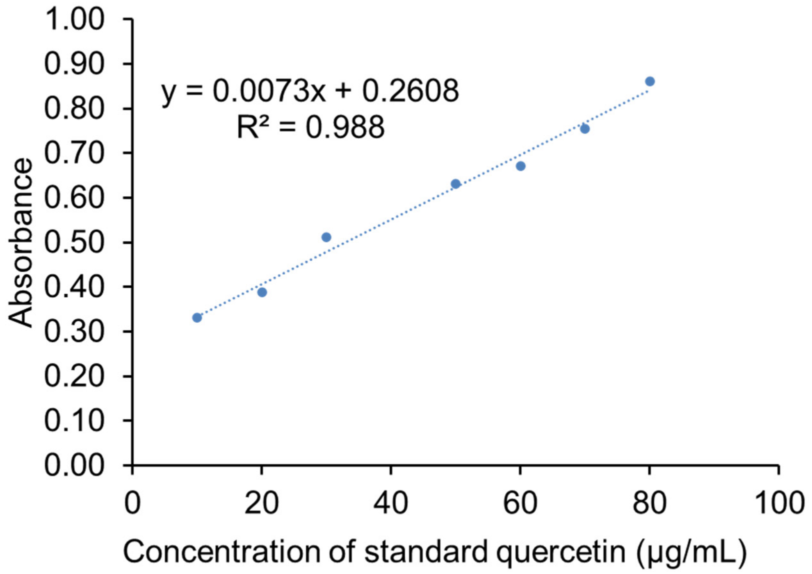

Figure S2: Quercetin Standard Graph.

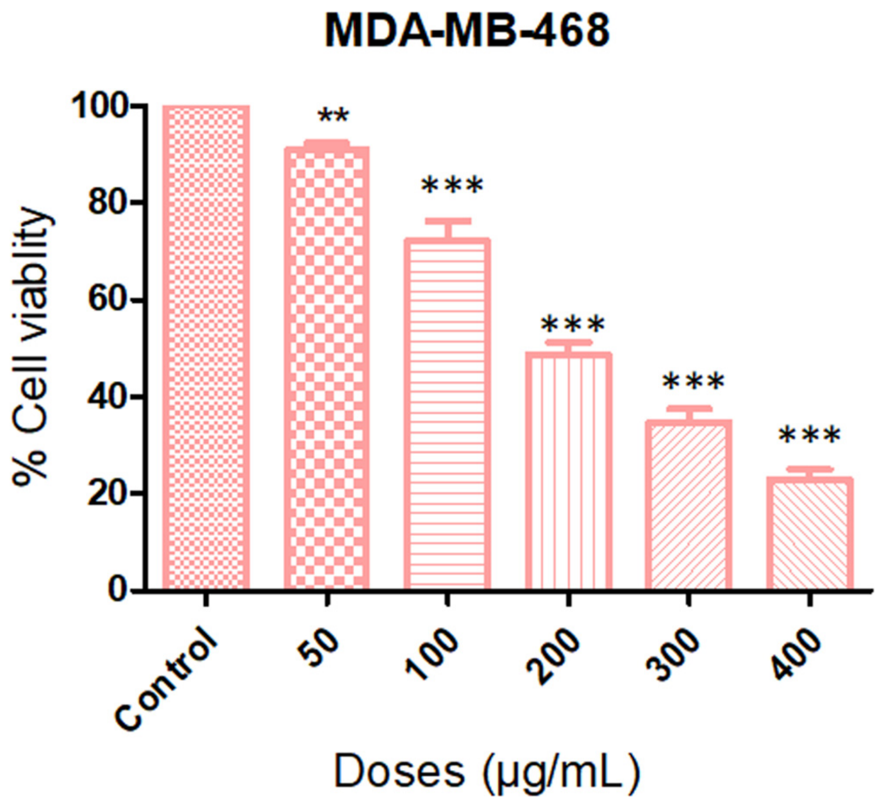

Figure S3. Cytotoxic test of *S. persica* bark extract on human TNBC breast cancer MDA-MB-468 cells. Cytotoxicity of bark extract was quantified as the percentage cell viability at 24 h. Values from a minimum of three independent experiments are shown as Mean ± SD, with **p<0.01 and ***p<0.001 in comparison to the control.
